# The immunomodulatory and antitumor properties of the bacterial metalloprotease Oligopeptidase A are mediated by TLR4/MyD88/TRIF and MAPK signaling pathways

**DOI:** 10.3389/fimmu.2025.1630886

**Published:** 2025-09-12

**Authors:** Priscila Silva, Gabrielli Novaes Silva, Filipe Menegatti Melo, Carolina de Amat Herbozo, Tarciso Almeida Sellani, Samanta Lopes Tomaz, Amanda Campelo L. De Melo, Larissa Reis Da Silva, Rodrigo Berzaghi, Marcelo F. M. Marcondes, Fellipe Bronze, Thaysa Paschoalin, Isaias Glezer, Adriana K. Carmona, Felipe Valença Pereira, Elaine Guadelupe Rodrigues

**Affiliations:** ^1^ Department of Microbiology, Immunology, and Parasitology, Paulista School of Medicine, Federal University of São Paulo (EPM-UNIFESP), São Paulo, Brazil; ^2^ Goethe University Frankfurt, Faculty of Medicine, Institute of Clinical Pharmacology, Frankfurt/Main, Germany; ^3^ David Geffen School of Medicine, Department of Microbiology, Immunology, and Molecular Genetics, Los Angeles, CA, United States; ^4^ Glaxosmithkline Brasil, Oncohematology, São Paulo, Brazil; ^5^ Sirio-Libanes Hospital, São Paulo, Brazil; ^6^ Department of Clinical Medicine, Faculty of Health Sciences, UiT The Arctic University of Norway, Tromsø, Norway; ^7^ Department of Biophysics, Paulista School of Medicine, Federal University of São Paulo (EPM-UNIFESP), São Paulo, Brazil; ^8^ Department of Biochemistry, Paulista School of Medicine, Federal University of São Paulo (EPM-UNIFESP), São Paulo, Brazil; ^9^ Department of Immunology and Microbiology, University of Colorado, Aurora, CO, United States

**Keywords:** B16F10, Oligopeptidase A (OpdA), bacterial metalloprotease, melanoma, immunoadjuvant, immunomodulation, toll-like receptor 4 (TLR4), MyD88/TRIF/MAPK

## Abstract

**Introduction:**

Immunosuppressive factors within the tumor microenvironment hinder effective antitumor immune responses and limit the efficacy of current immunotherapies. Immunomodulators offer an alternative by activating immune effectors. Proteases from various sources used as cancer therapy adjuvants have shown promise in inhibiting tumor growth. Our previous work showed that the bacterial metalloprotease arazyme has a strong in vivo antimetastatic effect in the B16F10-Nex2 murine melanoma model. Interestingly, heat-inactivated arazyme also exhibited antitumor properties dependent on an intact adaptive immune response, highlighting its immunomodulatory role. To assess whether this effect is unique to arazyme, we examined another bacterial metalloprotease, Oligopeptidase A (OpdA).

**Methods:**

OpdA was produced and purified. Endotoxin levels were measured. C57BL/6 mice received intravenous B16F10-Nex2 cells, followed by treatments with either active or heat-inactivated OpdA. Pulmonary nodules were counted. Immune cells involved in the response were characterized using FACS and depletion experiments. Cytokines were measured by ELISA and intracellular cytokine analysis. OpdA receptor activation was studied in bone marrow-derived cells from knockout and wild-type mice using inhibitors.

**Results:**

Heat-inactivated OpdA significantly reduced metastasis, dependent on tumor-specific CD4+ and CD8+ T cells and IFN-γ, both locally and systemically, with decreased IL-10 levels suggesting a proinflammatory environment. Treatment increased secretion of nitric oxide, IL-12p40, and TNF-α from bone marrow cells via enzymatic activity, involving MyD88/TRIF and MAPK pathways. Conclusion: OpdA shows potential as a tumor vaccine adjuvant, promoting antigen presentation and tumor-specific immune responses.

## Introduction

Melanoma is a serious skin cancer originating from melanocytes, accounting up 90% of skin cancer cases. It has proven resistant to chemotherapy, with some countries experiencing an annual growth rate of 3 to 7% (1). The introduction of immune checkpoint inhibitors and targeted therapies, such as BRAF/MEK inhibitors for BRAF-mutant melanoma, has greatly improved survival rates, including progression-free and overall survival. However, there is an urgent need to discover new treatments for patients whose melanoma does not respond to these current options (2).

The tumor microenvironment (TME) is a dynamic and intricate ecosystem where tumor cells interact with various immune and stromal cells. As tumors grow, the TME transitions from an initial pro-inflammatory, immune-activating phase to a later immunosuppressive state characterized by metabolic alterations and hypoxia. This change highlights the dual roles of key antitumor immune cells—such as T lymphocytes, natural killer cells, macrophages, dendritic cells, and myeloid-derived suppressor cells—which can either hinder or promote tumor growth depending conditions (3).

In recent years, immunomodulators have created new opportunities for cancer treatment. They boost the immune system’s ability to eliminate tumor cells by enhancing their interaction with standard cancer therapies. Immunomodulators are generally divided into categories such as checkpoint inhibitors, cytokines, agonists, or adjuvants (4).

Immunomodulators can activate key pathways of innate immunity, thereby enhancing a more effective adaptive immune response (5, 6).

Dendritic cells (DCs) are highly effective antigen-presenting cells, known for their ability to stimulate T cell immunity through TCD4^+^ and TCD8^+^ responses (7–9). The maturation of DCs begins with the recognition of antigens by pattern recognition receptors (PRRs), including Toll-like receptors (TLRs), which triggers cell activation. Once activated, DCs exhibit a reduction in phagocytic and endocytic receptors while increasing levels of co-stimulatory molecules (such as CD80, CD86, CD40, CD83, CD70, and OX40L). They also show enhanced expression of MHC I and II molecules and secrete pro-inflammatory cytokines and chemokines (like IL-12, IL-6, and TNF-α) (10–12). IL-12 plays a crucial role in activating Natural Killer (NK) cells and T cells, leading to the production of cytokines, primarily IFN-γ, which facilitates the generation of antigen-specific cytotoxic T lymphocytes (CTLs) and enhances NK cell cytotoxicity (13).

Toll-like receptors (TLRs) recognize pathogens through pathogen-associated molecular patterns (PAMPs) and endogenous danger signals, also known as danger-associated molecular patterns (DAMPs). By activating innate immunity, TLRs trigger the adaptive immune response. This has led to the idea that TLR ligands could be useful components in cancer vaccines (14).

The use of adjuvants has become especially important in antitumor vaccines because tumors often express “altered self” antigens that generally trigger a weaker immune response than pathogen antigens. Furthermore, immunoadjuvants that work well in pathogen vaccines may not be as effective in cancer vaccines (15). They can improve antigen uptake by antigen-presenting cells (APCs) by causing structural changes that promote gradual release and make the antigens more recognizable to the immune system (5).

Each TLR detects specific patterns; TLR4 (toll-like receptor 4) recognizes microbial lipopolysaccharides (LPS), which are part of the cell wall in gram-negative bacteria, as well as damage-associated molecules like heat shock proteins and high mobility group proteins, including HMGB1 and HMGN1 (High mobility group box 1 and High Mobility Group Nucleosome Binding Domain 1, respectively) (16, 17).

TLR4 activation by LPS starts when LPS binds to the LPS-binding protein (LBP). This enables LPS monomers to transfer to CD14, which is found in serum or on cell surfaces. Next, LPS is handed off to the accessory protein MD-2, forming the TLR4/MD-2 complex that promotes dimerization. This dimer then recruits two pairs of adapter proteins: TIRAP/MyD88 (myeloid differentiation factor 88) and TRAM/TRIF (the adapter containing the TIR domain that induces Interferon β). MyD88 helps recruit IRAK4 and IRAK2 (or IRAK1) kinases, which activate NF-kB and MAP kinase signaling pathways, leading to the production of pro-inflammatory cytokines like TNF-α and IL-6. Meanwhile, TRIF initiates a signaling cascade that activates IRF3, promoting the expression of IFN (type I interferon), IL-10, and RANTES (16, 18).

LPS enhances macrophage and monocyte activation by rapidly phosphorylating tyrosines, including key tyrosine kinase members like p53/56lyn and p58/64c-fgr, as well as both typical (PKCβ) and atypical (PKCζ) protein kinase C isoforms. These signals activate the MAPK pathway, which involves MAPK, MAPK kinase (MAPKK), and MAPK kinase kinase (MAPKKK). In human monocytic cells, LPS strongly activates all three components of this pathway (19).

Therefore, TLR4-ligands used as adjuvants in antitumor vaccines show great promise. MPLA (Monophosphoryl lipid A), a safer form of LPS, activates antigen-presenting cells (APCs), especially dendritic cells (DCs), by engaging TLR4. This activation boosts co-stimulatory molecule expression, promotes a Th1 immune response, and enhances IL-12 production (20). Additionally, the FDA has approved MPLA, a TLR4 agonist, as an adjuvant in therapeutic antitumor vaccines (21, 22).

Various proteases from different sources have shown promising results as therapeutic adjuvants in cancer treatment by inhibiting tumor growth. Notable examples include serine protease gingipains (23, 24), cysteine protease papain (25), bromelain from pineapple extract (26, 27), and the cysteine protease fastuosain (28).

Our previous study showed that intravenous injection of B16F10-Nex2 melanoma cells in mice, combined with intraperitoneal arazyme treatment, resulted in a significant reduction of lung nodules. Arazyme, a metalloprotease derived from *Serratia proteomaculans*, was characterized by Bersanetti et al. (29). Both its active and heat-inactivated forms exhibited antitumor effects, indicating these actions are independent of its proteolytic activity. Additionally, heat-inactivated arazyme displayed immunomodulatory effects on antigen-presenting cells (APCs), boosting the response of IFN-γ and CD8^+^ T-cells via the TLR4/MyD88/TRIF and MAPK pathways (30, 31).

To determine whether the antitumor and immunomodulatory effects were specific to arazyme, this study examined a different bacterial metalloprotease called Oligopeptidase A (OpdA). OpdA is a zinc-dependent enzyme belonging to the M3A subfamily, found in *Salmonella typhimurium* and *Escherichia coli*. It has a molecular weight of 77.1 kDa, as measured by gel filtration. This protease can hydrolyze small peptides and plays a role in various catabolic pathways in *E. coli*. It is also known for its ability to cleave bioactive peptides such as bradykinin and neurotensin (32–34).

## Material and methods

### Animals

Male C57Bl/6 (WT), MyD88^-/-^, TRIF^-/-^, TLR4^-/-^, TLR2^-/-^, IFN-γ-/-, and NOD-scid IL2Rγnull (NSG) mice, aged 6 to 8 weeks and kept in standard pathogen-free conditions, were acquired from the Center for Development of Experimental Models (CEDEME) and the Institute of Pharmacology and Molecular Biology (INFAR) at the Federal University of São Paulo (UNIFESP). The Animal Experimentation Ethics Committee (CEUA) at UNIFESP approved all conducted experiments (Protocol numbers 1882220815 and 5635020419).

### Cell lines and reagents

The cell line B16F10 was isolated from a spontaneous murine melanoma in C57Bl/6 mice (35) and obtained from Ludwig Cancer Research in São Paulo, Brazil. The B16F10-Nex2 subline was established at the Experimental Oncology Unit (UNONEX, at UNIFESP) and maintains the same metastatic and aggressive features as the original line (36). The cells were cultured in RPMI 1640 medium (Gibco) supplemented with 10% heat-inactivated fetal calf serum (FCS, Life Technologies), 10 mM HEPES (4-(2-hydroxyethyl)-1-piperazineethanesulfonic acid; Sigma Aldrich), 24 mM NaHCO_3_ (Sigma Aldrich), and 40 mg/mL gentamicin (Hipolabor Farmacêutica, MG, Brazil). Cultures were incubated at 37 °C with 5% CO_2_. IFN-γ was purchased from Peprotech, while lipopolysaccharides (LPS) and polymyxin B were obtained from Sigma Aldrich. The TLR-4 inhibitors (CLI-095), MyD88 inhibitor (Pepinh-MyD88), TRIF inhibitor (Pepinh-TRIF), and peptide control (Pepinh-Control), as well as SP600125 (JNK inhibitor), SB203580 (p38 inhibitor), and PD98059 (MAP/ERK inhibitor), were sourced from Invivogen, CA, USA. Peptides PAR1-AP (RLLFT-NH2) and PAR2-AP (LRGILS-NH2) were synthesized as previously described (37). The antibiotics (kanamycin and chloramphenicol) and IPTG (isopropyl β-D-thiogalactoside) were obtained from Sigma-Aldrich.

### Recombinant expression and purification of the OpdA enzyme

The expression vector pET-28 OpdA was constructed by inserting the OpdA gene into the pET28 plasmid, as previously described (32). Briefly, the OpdA gene was cloned via PCR from the total DNA of the *E. coli* strain DH5α using NcoI and XhoI restriction enzymes, resulting in a final construct with a poly-histidine tag at the C-terminus. Bacterial DNA was extracted using the PureLink Genomic DNA Purification Kit (Invitrogen). The PCR mixture contained 200 µM deoxynucleoside triphosphates, 2 mM MgCl_2_, 50 mM KCl, and 20 mM Tris-HCl (pH 8.4), 1 U pfu DNA polymerase, and 50 pmol of the primers (5’-GATAATCCATGGGCTCTAAAATTCTCCCGGAACATGTCG-3’ and 5’-ACCACCCTCGAGGCCCTTAATGCCGTAATGCTCCAGC-3’). The PCR reaction started with one cycle at 95 °C for 3 minutes, followed by 35 cycles at 95 °C for 1 minute, 72 °C for 2.5 minutes, and ended with 72 °C for 5 minutes. The PCR product was analyzed on a 1% agarose gel containing 1.0 µM ethidium bromide in TAE buffer (40 mM Tris-acetate and 1 mM EDTA, pH 8.0), purified using the Wizard SV Gel kit (Promega), and cloned into the pGEM-T Easy vector (Promega, Madison, USA). The cloned OpdA gene was excised from the plasmid by digestion with NcoI and XhoI (Invitrogen) and inserted into the EcoRI site of the pET28 vector, then sequenced.

For expression, *E. coli* BL21 (DE3) pLysS was transformed via heat shock with the expression vector pET-28 OpdA and grown under agitation at 37°C for 16 hours in Luria-Bertani medium supplemented with 50 µg/mL kanamycin and 50 µg/mL chloramphenicol. The bacteria pLysS/pET28-OpdA were re-inoculated in fresh medium containing the same antibiotic selection and grown until the culture density reached an A600 of 0.6. Subsequently, the expression of the OpdA recombinant was induced with 1 mM IPTG for 12 hours at 20°C. The bacterial cultures were centrifuged at 8,000 rpm for 20 minutes at 4°C, re-suspended in 20 mM Na_2_HPO_4_, 300 mM NaCl, and 20 mM imidazole, pH 8.0, and lysed by pressure in a French Pressure Cell Press (model FA-078, Thermo Spectronic). The suspension was incubated for 20 minutes under agitation with 20 mM MgSO_4_ and barnase (2.4 units/mL). The solution was centrifuged to remove the bacterial debris for 15 minutes at 14,000 rpm at 4°C, and the supernatant was filtered through a 0.45 µm filter. The solution was loaded into a Ni-Sepharose column (HisTrapTM HP Columns – GE Healthcare) coupled with the ÄKTA purifier System (GE Healthcare). The column was washed with a buffer containing 20 mM Na_2_HPO_4_ and 300 mM NaCl, and then OpdA was eluted using segmented step elution with 50 mM and 100 mM imidazole. The recombinant protein was desalted using a PD-10 column (Amersham Pharmacia Biotech) and analyzed by SDS-PAGE electrophoresis (10%), stained with silver, and further assessed by western blotting (**Supplementary Figure 1A**).

### Heat-inactivation and activity of OpdA metalloprotease

The hydrolysis of the fluorogenic peptidyl substrate Abz-GFSIFRQ-EDDna was measured using a HITACHI F-2000 spectrofluorometer with excitation and emission wavelengths of 320 nm and 420 nm, respectively, at 37°C in a 50 µM Tris-HCl buffer, pH 7.4 (32). The substrate solution was kept in a thermostatically controlled chamber for 5 minutes before adding 20 µM recombinant OpdA. The slope was converted to moles of hydrolyzed substrate per minute based on the fluorescence curves of standard peptidase solutions before and after complete hydrolysis. The data were used to plot the kinetic curve of OpdA enzymatic activity (**Supplementary Figure 1E**). OpdA was heated for 45 minutes at 65 °C to achieve heat inactivation. The structural integrity of the heat-inactivated protease was confirmed by SDS-PAGE electrophoresis (10%), stained with silver (**Supplementary Figure 1B**). The loss of proteolytic activity was demonstrated by the absence of activity on the fluorogenic substrate Abz–GFSIFRQ–EDDnp under the same conditions described (**Supplementary Figure 1D**).

### Purified OpdA identification using western blotting

OpdA was separated using SDS-PAGE and then electro-transferred onto a nitrocellulose membrane. The membrane was incubated for 16 hours at 4°C with in-house prepared murine anti-OpdA primary antibodies. After this, it was treated with a peroxidase-conjugated anti-mouse IgG secondary antibody for 90 minutes at 20°C. Finally, the blot was incubated with SuperSignal Western Blot Enhancer (Thermo Scientific) and visualized using the UVITEC System (UVITEC Cambridge) (**Supplementary Figure 1A**).

### OpdA digestion by proteinase K

OpdA (100 µg/mL) was digested with 50 µg/mL proteinase K (Sigma Aldrich) and 2.5 mM CaCl_2_ in RPMI 1640 medium. The sample was incubated for 45 minutes at 37°C with agitation, and the digestion was confirmed by SDS-PAGE 10% with silver staining (**Supplementary Figure 1C**).

### Measurement of endotoxin contaminants

The endotoxin contaminants in OpdA samples were measured using the Endpoint Chromogenic LAL (*Limulus amebocyte* lysate) assay kit (Lonza) following the manufacturer’s instructions.

### Cell proliferation assay

Cell proliferation was assessed by measuring mitochondrial dehydrogenase enzyme activity using the MTT (3-(4,5–dimethyl–2–thiazolyl)-2,5-diphenyl-2H-tetrazolium bromide) cell assay. B16F10-Nex2 cells (4x10^3^) were grown in 96-well plates, and after 24 hours, they were incubated for an additional 24 or 48 hours with various concentrations of OpdA. Next, the cells were incubated with 10µL of MTT (5 mg/mL) for 3 hours at 37°C in 5% CO_2_, followed by the addition of 100 µL SDS in 0.01 M HCl. The plates were analyzed using Spectramax M2 equipment (Molecular Devices) at 570 nm with a 650 nm reference filter (**Supplementary Figure 3**).

### OpdA treatment in the metastatic melanoma murine model

To analyze the effect of OpdA on B16F10-Nex2 melanoma development *in vivo*, male C57BL/6, NSG (Nod Scid gamma null), and MyD88^-/-^ mice were inoculated via the caudal vein with 5x10^5^ B16F10-Nex2 cells in 100µL of RPMI medium without FCS. One day after inoculation, these animals received intraperitoneal injections of either active or heat-inactivated OpdA (50µg) or PBS (control) on alternate days for two weeks. Afterwards, the melanotic lung nodules were visually quantified using an SMZ 745T stereoscopic microscope (Nikon), and images were captured with a DS-U3 digital camera (Nikon) attached to the system.

The adaptive immune response induced by OpdA treatment was assessed in male C57BL/6 and IFN-γ KO mice, divided into three groups (control and treated with active or inactive OpdA), each consisting of three animals. Mice were challenged intravenously with 1.5 x 10^5^ B16F10-Nex2 cells inoculated via the tail vein and underwent the same treatment protocol described.

After this period, the animals were anesthetized intraperitoneally with ketamine/xylazine (100 mg/kg and 10 mg/kg, respectively) for blood collection via cardiac puncture, then euthanized with 300 mg/kg and 30 mg/kg of ketamine/xylazine, respectively. The lungs, whole blood, draining lymph nodes, and spleens were collected from each animal for later analysis of effector cells and cytokines.

### *Ex vivo* stimulation of tumor-specific lymphocytes derived from splenocytes and lymph node cells

Spleens and draining lymph nodes (cervical, axillary, and inguinal) were macerated and filtered through a cell strainer (Corning) for cell isolation. Erythrocytes were removed using a hemolytic buffer (8.3 g/L NH_4_Cl, 1 g/L NaHCO_3_, and 0.02 g/L disodium EDTA) for one minute at 4°C. To neutralize the hemolytic buffer, three washes with 10% FCS-RPMI medium were performed. Following centrifugation, the supernatant was discarded, and the cells were resuspended in 10% FCS-RPMI medium. The viable cells were counted with trypan blue dye. Cells were cultured in 24-well plates for 96 hours, either with or without tumor lysate, to analyze and quantify tumor-specific CD4^+^ and CD8^+^ T lymphocytes that produce IFN-γ and IL-10. *Ex vivo* re-stimulation was done at a 10:1 ratio of immune cells to tumor cells per well, using tumor lysate equivalent to 10^5^ B16F10-Nex2 cells. The tumor lysate was prepared by lysing 5×10^6^ B16F10-Nex2 cells (in 2.5 mL of RPMI) through 8 cycles of freezing in liquid nitrogen and heating in a 37°C water bath, followed by centrifugation at 3,000 rpm, discarding the pellet, and applying 50 µL per well.

### Analysis of intracellular production of IFN-γ and IL-10 by CD4^+^ and CD8^+^ T lymphocytes

Splenocytes (5 x 10^6^) were restimulated *ex vivo* in 96-well plates with tumor lysate equivalent to 5 x 10^5^ tumor cells (ratio 10 splenocytes to 1 tumor cell) in 200 µL of RPMI/10% FCS medium. The splenocytes were stimulated for 96 hours, and during the last 5 hours, 10 µg/mL of Brefeldin A (Sigma Aldrich) was added. The cells were collected, centrifuged, and their Fc receptors blocked with a pool of sera from normal C57Bl/6 mice, inactivated at 56°C and diluted 1:30 in PBS containing 1% BSA (Sigma Aldrich) for 1 hour at 4°C. They were then washed and labeled with 50 µL of monoclonal antibodies conjugated to fluorescent molecules: anti-CD3ϵ-eFluor450 (clone 145-2C11, eBioscience), anti-CD4-FITC (clone RM4-5, eBioscience), and anti-CD8α-PerCPCy5.5 (clone 53-6.7, eBioscience), diluted 1:50 in PBS containing 1% BSA (Sigma Aldrich), for 1 hour at 4°C in the dark. Next, they were washed and fixed with 2% paraformaldehyde for 15 minutes. Cells were permeabilized with a buffer composed of 0.5% saponin (Sigma Aldrich) in PBS for 10 minutes at room temperature. The reaction was then blocked as above. Anti-IFN-γ-biotin (clone XMG1.2, BD Pharmingen) and anti-IL-10-PE (clone JES5-16E3, BD Pharmingen) monoclonal antibodies were diluted 1:50 in permeabilization buffer and incubated with the cells for 1 hour at 4°C. After washing twice with permeabilization buffer, the cells were incubated with streptavidin-FITC (BD Pharmingen) for 1 hour at 4°C, diluted in the same buffer. All samples underwent two additional washes with permeabilization buffer and PBS before being fixed with 500 µL of 2% paraformaldehyde in PBS. The samples were acquired on the FACSCanto II flow cytometer (BD Biosciences) and analyzed using FlowJo software (Tree Star). Positive controls for IFN-γ production were stimulated for 5 hours with PMA at 50 ng/mL and ionomycin at 1 µg/mL (both Sigma-Aldrich). Controls for IL-10 production were stimulated for 96 hours with anti-CD3 and anti-CD28 (both at 1 µg/mL, BD Biosciences), and in the last 48 hours, IL-2 (20 ng/mL, PeproTech) and IL-4 (50 ng/mL, PeproTech) were added.

### *In vivo* depletion of CD4^+^ and CD8^+^ T lymphocytes

C57Bl/6 mice received intraperitoneal treatments of two doses (3 days before and 7 days after tumor cell challenge) of 0.5 mg of anti-CD4 monoclonal antibody (clone GK1.5), or 1 mg of anti-CD8 (clone YTS), both prepared and purified in-house by Luiz S. Silva. Three days after the initial antibody treatment, the animals were inoculated intravenously with 1.5x10^5^ B16F10-Nex2 cells. On the day following the challenge, they were treated with 50 µg of active or inactive OpdA, administered every other day for 14 days. On the 15th day after the challenge, the lungs were harvested for nodule counting.

### Differentiation of murine bone marrow progenitors

Bone marrow-derived DCs (BMDCs) were differentiated from C57Bl6, TLR4^-/-^, TLR2^-/-^, TRIF^-/-^, or MyD88^-/-^ mice as described previously (38). BMDCs were cultured in Petri dishes (100 mm, Corning) in RPMI/10% FCS, supplemented with non-essential amino acids (1X), 50 µM 2-mercaptoethanol (all from Gibco), and 30 ng/mL murine rGM-CSF (PrepoTech), 10 mL per femur. On the 4th day, complete medium was added to the cultures, and after 7 days, numerous non-adherent, typical DCs were obtained.

### Identification of activated bone marrow-derived cells by flow cytometry

BMDCs were cultured in 6-well plates (2x10^6^ cells per well) and stimulated with 50 µg/mL of either active or heat-inactivated OpdA for 48 hours. The cells were washed twice with PBS containing 1% BSA, and Fc receptors were blocked as described above. BMDCs were then incubated with antibodies diluted 1:60 in PBS with 1% BSA for one hour at 4 °C, washed twice with PBS, and then fixed with 2% paraformaldehyde. BMDC phenotypes were assessed using APC-labeled anti-CD11c, PE-labeled anti-CD80, PE-labeled anti-CD86, and FITC-labeled anti I-Ab, all produced by eBioscience/Thermo Fisher Scientific. Data acquisition was performed with a FACS Canto II (BD Biosciences), and population analyses were conducted using FlowJo Software (Tree Star, CA, USA).

### Cytokine and nitric oxide quantification

BMDCs were cultured in 96-well plates (1x10^5^ cells per well) with varying concentrations of active or inactive OpdA, 200 U/mL IFN-γ, 200 ng/mL LPS, and, in some wells, with 20 µg/mL polymyxin B or OpdA digested with 50 µg/mL proteinase K for 48 hours. Afterwards, the culture supernatant was collected, and nitric oxide (NO) levels were measured using Griess reagent (39). Tumor necrosis factor-alpha (TNF-α) and IL-12p40 were quantified by ELISA, and a TMB substrate solution (both from BD Bioscience) was used for detection. Both ELISA and NO measurements were read with a Spectramax M2 plate reader (Molecular Devices), with NO at 540 nm and cytokines at 450 nm, applying correction at 570 nm.

Cytokines were also measured in the culture supernatant of splenocytes and lymph node cells, as well as in sera and lung homogenates, all collected on the 15th day after tumor cell inoculation, from animals treated or not with OpdA. Kits for the cytokines IFN-γ and IL-10 (BD Bioscience) were used according to the manufacturer’s instructions. At the end of the protocols, plates were washed six times with Wash buffer, and 50 μL of ELISA chromogen substrate containing hydrogen peroxide (BD Pharmigen^®^ TMB Substrate BD 555214) was added. Plates were kept in the dark for an additional 30 minutes, and after this period, 50 μL of Stop Solution (4N sulfuric acid) was added. The reaction was read at a wavelength of 450 nm (reference 630 nm) using a SpectraMax M2e (Molecular Devices). The cytokine concentration was calculated from the standard curve of concentration versus absorbance graph, using Microsoft Excel.

### Statistical analysis

Statistical analysis was performed using GraphPad Prism software (GraphPad Software, La Jolla, CA, USA) as described in each figure. In all studies, a *p-value <*0.05 was considered statistically significant.

## Results

### The antitumor activity of inactive OpdA depends on the presence of IFN-γ, and CD8^+^ T lymphocytes

The recombinant OpdA was produced using a highly efficient process, resulting in a high-purity product that was identified by an in-house prepared anti-OpdA polyclonal antibody (**Supplementary Figure 1A**). This metalloprotease demonstrated proteolytic activity on a specific substrate that exhibited intramolecular fluorescence suppression (**Supplementary Figures 1D, E**). The heat-inactivated metalloprotease maintained the structural integrity of the active enzyme (**Supplementary Figure 1B**) and showed no proteolytic activity on the same substrate (**Supplementary Figure 1D**).

**Figure 1:**
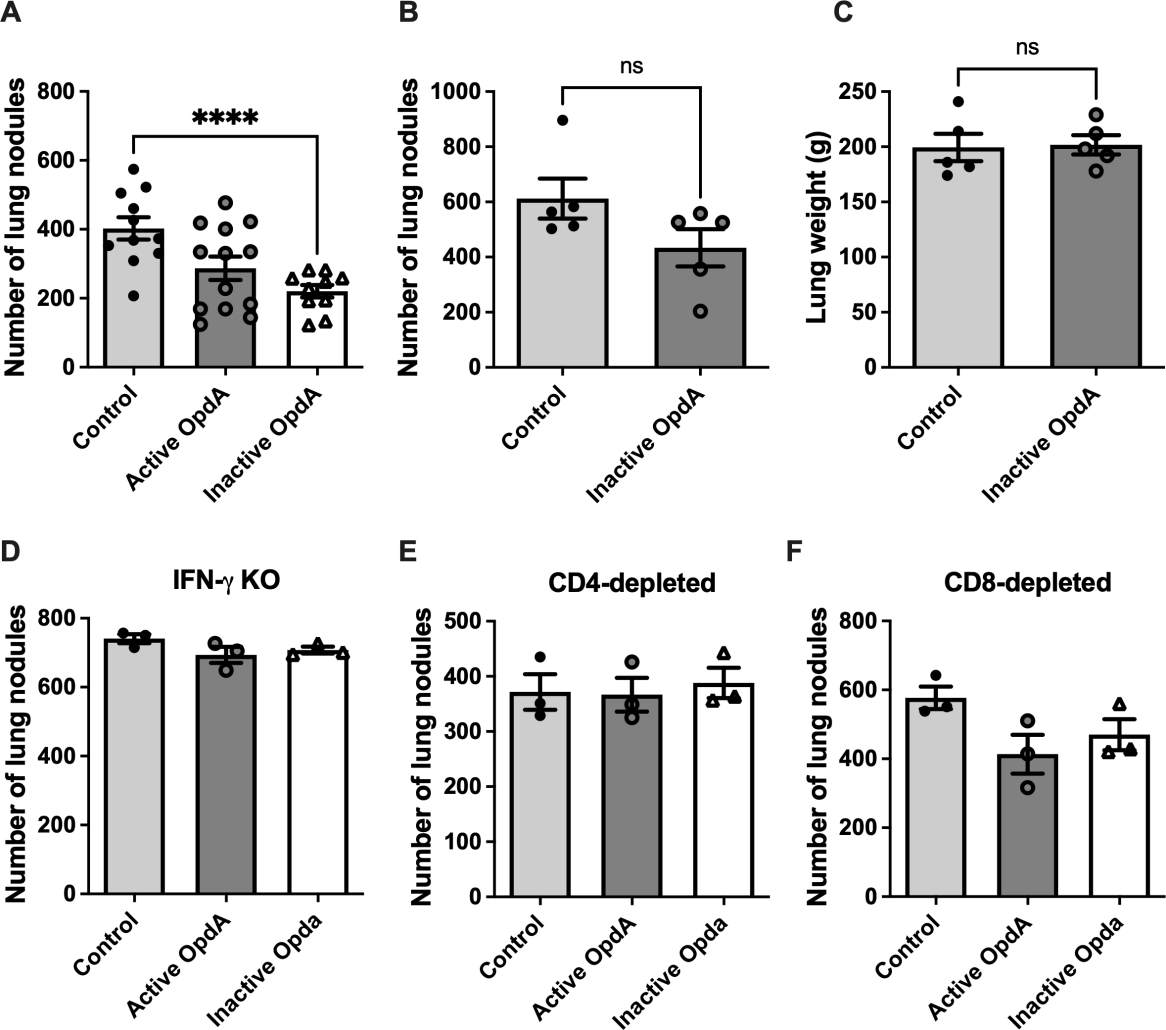
The adaptive immune system contributes to the *in vivo* antitumor effect of heat-inactivated OpdA. **(A)** Male C57Bl/6 mice (n=11 or 13) were injected intravenously with 5x10^5^ B16F10-Nex2 melanoma cells and treated intraperitoneally with 50 µg of either active or heat-inactivated OpdA, or PBS (control) on alternate days for two weeks. Melanotic pulmonary nodules were then counted using an inverted microscope. Individual animal data are shown, with the average (bars) and ± SD (vertical lines); **(B)** NSG mice (n=5) received the same treatment as in **(A)** with heat-inactivated OpdA^;^ **(C)** average lung weight (± SD) of NSG mice; **(D)** IFN-γ^-/-^ mice (n=3) were treated as described in **(A, E, F)** C57Bl/6 mice (n=3 per group) were given anti-CD4 or anti-CD8 antibodies intraperitoneally (three days before and seven days after tumor cell inoculation) in addition to the same treatment described in **(A)**. n.s., non-significant; ****p < 0.0001, analyzed by one-way ANOVA followed by the Tukey test. This represents one of two independent experiments.

To investigate the *in vivo* antitumor activity of OpdA, male C57Bl/6 mice were intravenously inoculated with B16F10-Nex2 cells and treated with either active or inactive OpdA. Mice receiving inactive OpdA showed fewer metastatic lung nodules than the control group (PBS-treated mice) (**Figure 1A**, **Supplementary Figure 2**), suggesting a potential indirect and non-specific effect of OpdA on tumor growth, which was further confirmed by the MTT cell viability assay (**Supplementary Figure 3**).

To determine whether the antitumor response triggered by inactive OpdA depends on a functional adaptive immune system, immunodeficient mice (NSG) were injected with B16F10-Nex2 cells and treated with inactive OpdA. The antitumor effect of inactive OpdA was lost in these mice; in fact, the control and treated groups had a similar number of lung nodules (**Figure 1B**) and comparable lung weights (**Figure 1C**). This shows that the antitumor response from inactive OpdA relies on an active adaptive immune response.

To assess how key effectors in the adaptive immune system contribute to the protective anti-tumor response triggered by OpdA, we used genetically modified animals lacking IFN-γ and performed *in vivo* depletion of CD4^+^ and CD8^+^ T lymphocytes using specific monoclonal antibodies. Notably, it was observed that the absence of the IFN-γ gene caused both active and inactive OpdA-treated animals to develop similar numbers of pulmonary nodules as the PBS-treated control group (**Figure 1D**). A similar outcome was seen in CD4- and CD8-depleted animals (**Figures 1E and F**, respectively), suggesting that these immune components are essential for the protective anti-tumor response mediated by either active or inactive OpdA.

**Figure 2:**
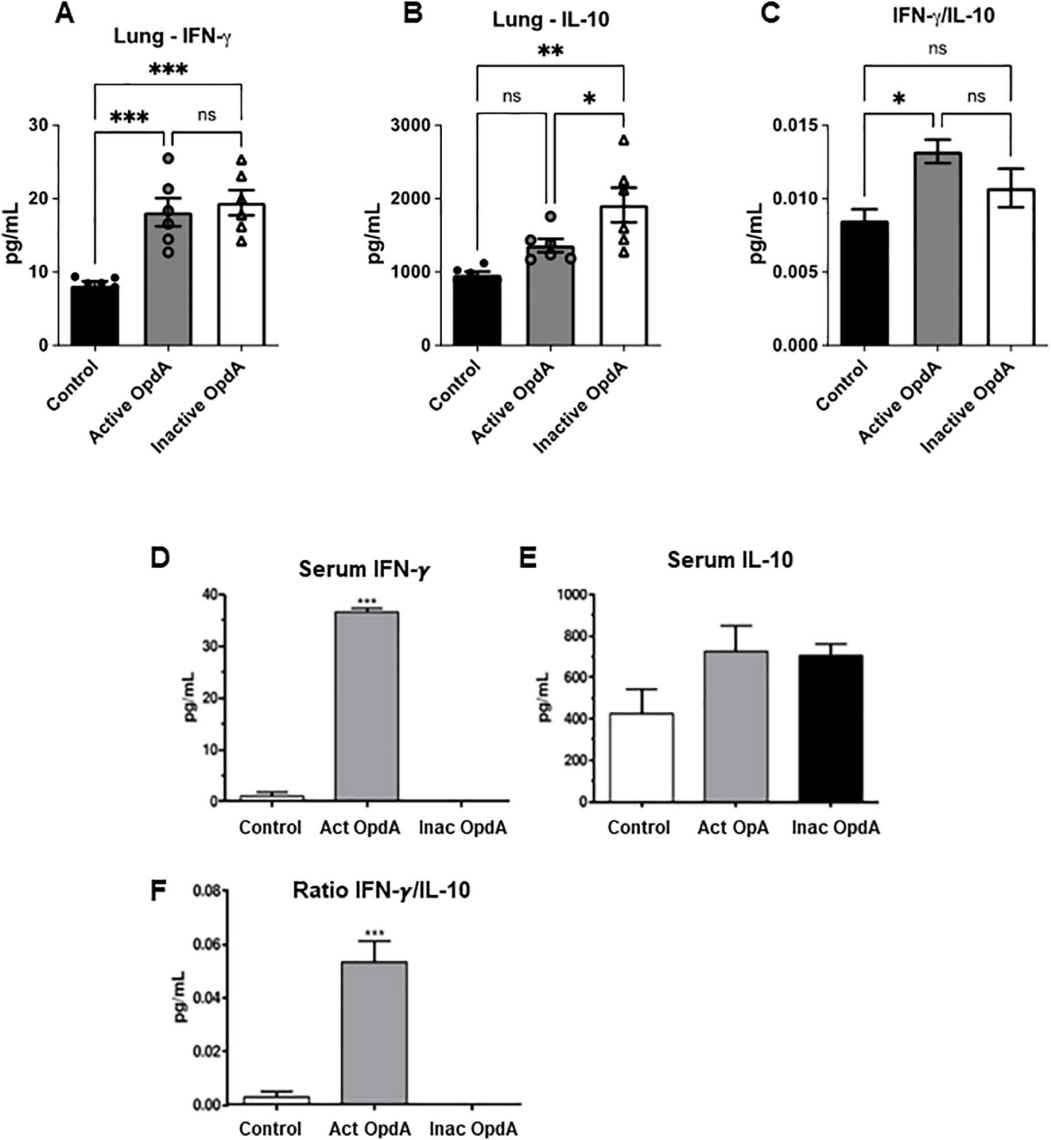
Quantification of IFN-γ and IL-10 levels in the lungs and serum of mice treated with either active or inactive OpdA in the metastatic melanoma model. C57Bl/6 mice (n=6 per group) received an inoculation of 1.5 x 10^5^ B16F10-Nex2 cells via the tail vein. On the first day after the challenge, the mice were treated intraperitoneally with 50 µg of either active or inactive OpdA or PBS (Control), on alternate days for two weeks. **(A)** IFN-γ levels in lung homogenates; **(B)** IL-10 levels in lung homogenates; **(C)** The IFN-γ/IL-10 ratio in lung homogenates; **(D)** Measurement of IFN-γ in pooled serum; **(E)** Measurement of IL-10 in pooled serum; **(F)** The IFN-γ/IL-10 ratio in pooled serum for each group. N.S., non-significant; ***p < 0.0001; **p < 0.001; *p < 0.05, determined by one-way ANOVA with Tukey’s test relative to the control group. Individual animal data are displayed, with mean (bars) and ± SD (vertical lines) indicated; the result is from one of two independent experiments.

We also measured the levels of IFN-γ and IL-10 in the lung homogenates and serum of mice treated with OpdA. In the lung homogenates, there was a notable increase in IFN-γ production in both the active and inactive OpdA-treated groups compared to the control group (**Figure 2A**), while IL-10 levels significantly increased only in the inactive recombinant-treated group (**Figure 2B**). The ratio of IFN-γ to IL-10, which indicates a proinflammatory antitumor protective response, was significantly higher in the active OpdA group. However, the inactive OpdA group also showed a tendency toward an increase (**Figure 2C**).

Serum from each group was pooled and analyzed for IFN-γ and IL-10 levels. The group treated with active OpdA showed a significant increase in IFN-γ levels (**Figure 2D**), while IL-10 production did not differ significantly across the groups (**Figure 2E**). The ratio of IFN-γ to IL-10 was elevated only in the group receiving active OpdA (**Figure 2F**).

We also examined the presence of tumor-specific CD4^+^ and CD8^+^ T lymphocytes that produce IFN-γ and IL-10 in the spleens and lymph nodes of OpdA-treated animals.

**Figure 3:**
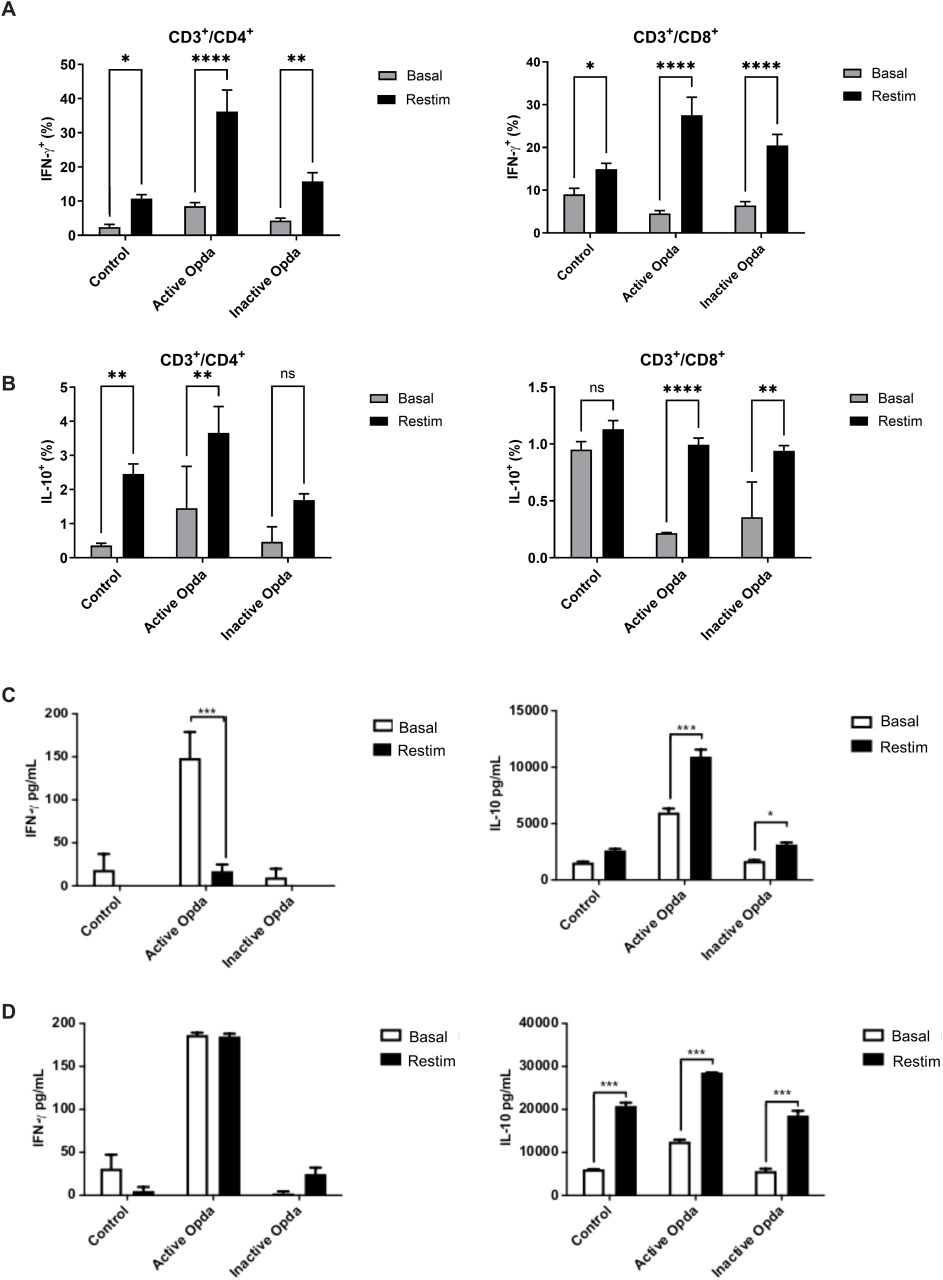
Measurement of intracellular and secreted IFN-γ and IL-10 by tumor-specific T lymphocytes from animals treated with either active or inactive OpdA. C57Bl/6 mice (n=3 per group) were injected via the tail vein with 1.5 x 10^5^ B16F10-Nex2 cells. On the first day after the challenge, the mice received 50 µg of either active or inactive OpdA or PBS (control) intraperitoneally every other day for a total of 15 days. Splenocytes were harvested, and 10^6^ cells were distributed into 24-well plates and cultured for 4 days, either restimulated (Restim) or left at baseline (Basal) with B16F10-Nex2 cell lysate at a 10:1 ratio (splenocytes: tumor cells); flow cytometry was performed at the end for analysis. **(A)** Percentage of IFN-γ^+^ cells among CD3^+^/CD4^+^ or CD3^+^/CD8^+^ tumor-specific T lymphocytes; **(B)** Percentage of IL-10^+^ cells among CD3^+^/CD4^+^ or CD3^+^/CD8^+^ tumor-specific T lymphocytes. Mice received the same treatments as outlined, after which splenocytes and lymph nodes were collected. Two million cells were distributed into 96-well plates and cultured as described. Cytokines produced were then quantified through ELISA from the culture supernatant. **(C)** Secretion of IFN-γ and IL-10 from tumor-specific lymph node cells; **(D)** Secretion of IFN-γ and IL-10 from tumor-specific splenocytes. ****p< 0,00001, ***p < 0.0001, **p < 0.001, *p < 0.05 determined via One-way ANOVA, followed by Tukey’s test. Bars represent the average with ± SD (vertical lines); one of three independent experiments is shown.

In mice treated with active OpdA, there was a significant increase in CD4^+^/IFN-γ, CD4^+^/IL-10, and CD8^+^/IFN-γ lymphocytes compared to those treated with PBS (**Figures 3A, B**). Conversely, mice receiving inactive OpdA only showed a statistically significant decrease in CD8^+^/IL-10 lymphocytes compared to the control group (**Figure 3B**).

The evaluation of IFN-γ and IL-10 levels in the culture supernatant, following *ex vivo* stimulation of cells from tumor-draining lymph nodes with tumor antigens, indicated that animals given active OpdA produced significantly higher levels of IFN-γ and IL-10 even without tumor stimuli compared to the control group. This suggests that these cells were likely pre-activated at that site. When stimulated with tumor antigens, a notable increase in IL-10 secretion was observed (**Figure 3C**). Cells from animals treated with inactive OpdA showed a significant increase in IL-10 secretion upon stimulation with tumor antigens (**Figure 3C**). Similar results were seen in splenocytes, where, in the absence of tumor antigen stimulation, their behavior mirrored that of the cells from the lymph nodes (**Figure 3D**). All groups showed a significant rise in IL-10 production upon re-stimulation with tumor antigens, especially in cells from animals treated with active OpdA. However, whether in lymph node cells or splenocytes, animals treated with active OpdA had a higher IFN-γ/IL-10 ratio compared to the other groups (**Figures 3C, D**).

The findings indicate that the antitumor protective effect of OpdA relies on a functioning adaptive immune system, as well as the presence of IFN-γ, CD4^+^, and CD8^+^ T cells.

### OpdA activates BMDCs through the production of IL-12, TNF-α, and NO

To gain a deeper understanding of how OpdA’s immunomodulatory mechanism induces a protective antitumor immune response, we examined the effect of the metalloprotease on BMDCs.

**Figure 4:**
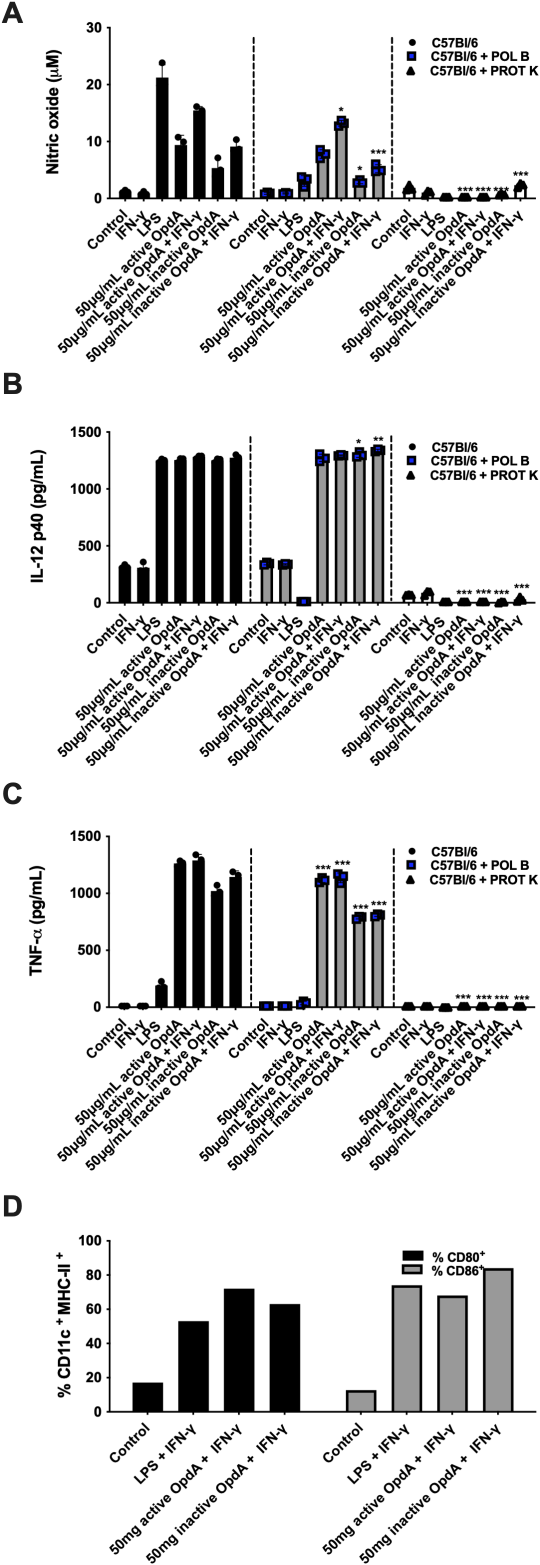
BMDCs are activated by either active or heat-inactivated OpdA. Bone marrow-derived dendritic cells (BMDCs) were generated from bone marrow progenitors of C57Bl/6 mice using GM-CSF. A total of 1x10^5^ cells were placed in U-bottom plates and stimulated with 50 μg/mL of active or heat-inactivated OpdA, with or without digestion by Proteinase K (PROT K, 50 μg/mL), along with IFN-γ (200 U/mL) and/or LPS (200 ng/mL) for 48 hours. In some wells, Polymyxin B (POL B, 20 μg/mL) was included, as indicated. Nitric oxide (NO) levels were measured in the culture supernatants using Griess reagent, and cytokine levels were determined via ELISA. **(A)** NO; **(B)** IL-12 (p40); and **(C)** TNF-α. Bars represent the mean ± SD from triplicate samples, with data from one of two independent experiments shown. **(D)** The expression of CD80 and CD86 was evaluated by FACS in CD11c^+^/MHCII^+^ positive cells. Statistical significance was analyzed using two-way ANOVA with the Bonferroni test, comparing controls without Proteinase K or Polymyxin B to the same stimuli, with *p < 0.05, **p < 0.001, and ***p < 0.0001.

BMDCs exposed to either active or inactive OpdA produced NO, especially when IFN-γ was present, although this was less effective than LPS stimulation (**Figure 4A**). There was a significant increase in IL-12p40 production, similar to levels seen with LPS stimulation, and IFN-γ did not affect this process (**Figure 4B**). BMDCs treated with active or inactive OpdA released higher amounts of TNF-α, whereas LPS was less effective (**Figure 4C**). Incubation with OpdA in the presence of IFN-γ led to increased expression of CD80 and CD86, yielding results comparable to LPS (**Figure 4D**). These findings show that OpdA activates BMDCs to promote an antitumor immune response.

The recombinant OpdA is expressed in *E. coli*, suggesting that the purified metalloprotease may contain endotoxin contaminants, mainly LPS, a potent TLR-4 ligand with immunomodulatory activity in antigen-presenting cells. To rule out possible BMDC activation caused by contaminant LPS, we treated *in vitro* cultures with polymyxin B, and stimulated BMDC cultures with proteinase K-treated OpdA. Polymyxin B is a cationic cyclic lipopeptide that binds to the lipid A portion of LPS and blocks its interaction with TLR-4 in BMDCs (40). Proteinase K is a serine protease that fully cleaves OpdA (41, **Supplementary Figure 1C**). The presence of polymyxin B (20 μ/mL) in the assay significantly inhibited the stimulatory effect of LPS as expected, and it is evident that, although there was a partial reduction in the production of NO and TNF-α (but not IL-12p40), the activation induced by OpdA in the BMDCs remains largely intact. Conversely, when BMDCs were stimulated with OpdA treated with proteinase K, the production of cytokines and NO was completely abolished, indicating that the stimulation of BMDCs depended on the presence of OpdA in the recombinant preparation, since LPS is resistant to proteinase K but the protease fully degraded OpdA (**Supplementary Figure 1C**).

### Endotoxin contamination cannot activate BMDCs

**Figure 5:**
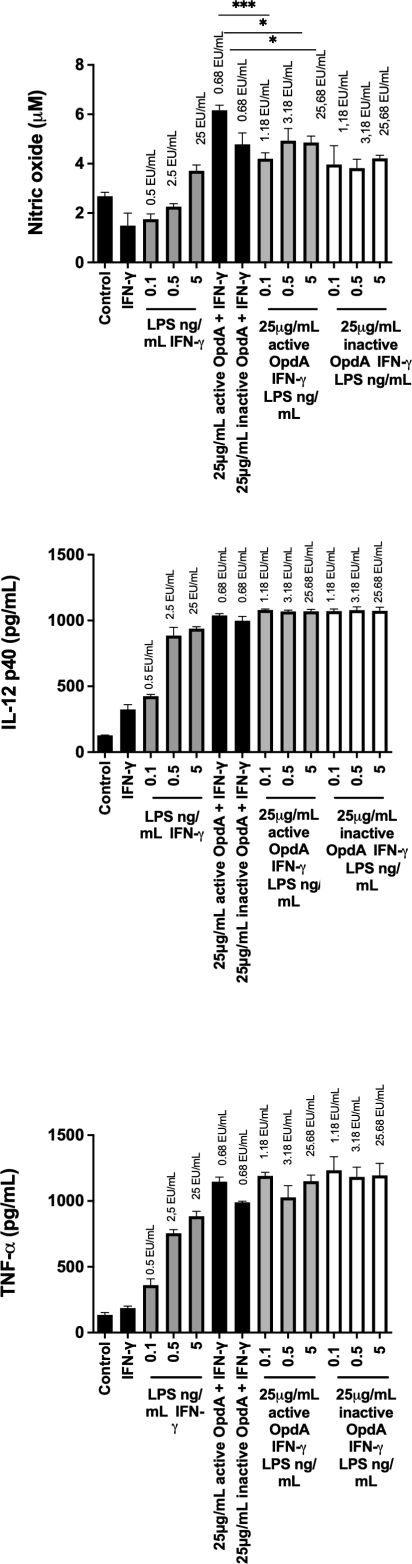
Effect of different concentrations of OpdA and LPS on the production of NO, IL-12p40, and TNF-α by BMDCs. BMDCs were derived from bone marrow progenitors of C57Bl/6 mice using GM-CSF. 1x10^5^ cells were plated in U-bottom plates and stimulated for 48 hours with active or inactive OpdA (25 μg/mL), IFN-γ (200 U/mL), various LPS concentrations, or combined OpdA and LPS, as described. The culture supernatants were tested for **(A)** NO using the Griess reagent, **(B)** IL-12p40, and **(C)** TNF-α via ELISA. The EU/mL values above each bar show LPS levels measured by the LAL assay. Bars indicate the mean and ± SD from triplicate samples. This is one of two independent experiments. *p < 0.05 and ***p < 0.0001, assessed by one-way ANOVA with Tukey’s test, comparing samples with 25 μg/mL active or heat-inactivated OpdA alongside IFN-γ.

Next, we evaluated how endotoxin residues in the recombinant OpdA preparation might influence the immunomodulatory properties of the protease. In a BMDC activation assay, we measured endotoxin levels in wells containing LPS, OpdA, or both, using a *Limulus amebocyte* Lysate (LAL) assay. The results showed that adding 0.1 ng of LPS to BMDCs equals 0.5 EU (endotoxin units), while adding 25 µg of OpdA contains 0.68 EU (**Figure 5**).

**Figure 5** shows that BMDCs incubated with 25 µg/mL of either active or heat-inactivated OpdA in the presence of IFN-γ produced significant levels of NO, IL-12p40, and TNF-α. In contrast, 0.1 ng/mL of LPS (0.5 EU/mL) did not induce NO, IL-12p40, and TNF-α as effectively as 25 µg of OpdA (0.68 EU). Additionally, the higher concentration of LPS tested, 5 ng/mL (25 EU/mL), also failed to activate BMDCs with the same efficiency as 25 µg/mL OpdA (0.68 EU/mL).

Another potential scenario involves a synergistic effect between LPS and OpdA. To evaluate this, BMDCs were stimulated with 25 μg/mL of either active or heat-inactivated OpdA along with increasing concentrations of LPS (from 0.1 to 5 EU/mL). Results showed that none of the tested LPS concentrations increased the production of NO, IL-12p40, or TNF-α, thereby ruling out the possibility of a synergistic interaction between LPS and OpdA (**Figure 5**).

These results support our previous findings, indicating that although the recombinant OpdA preparation contains low levels of contaminating endotoxin residues, these are not responsible for the immunomodulatory effect of OpdA on antigen-presenting cells.

### TLR4 is required for the activation of BMDCs by OpdA

**Figure 6:**
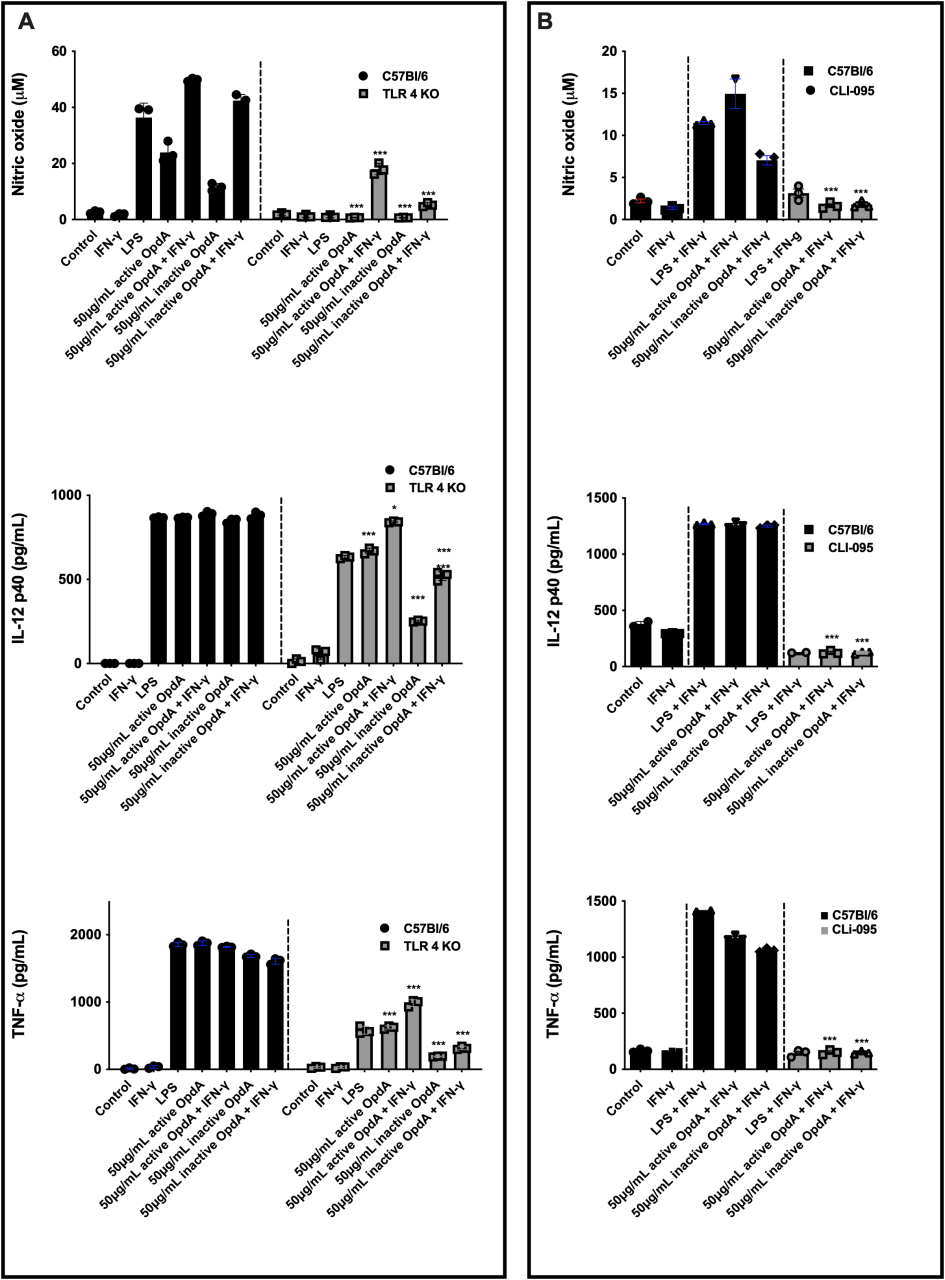
Active and heat-inactivated OpdA activate BMDCs through TLR4. **(A)** BMDCs were differentiated from bone marrow progenitors of C57Bl/6 and TLR4^-/-^ mice in the presence of rGM-CSF. 1x10^5^ cells were added to U-bottom plates and stimulated with active or heat-inactivated OpdA (50 μg/mL), IFN-γ (200 U/mL), and LPS (200 ng/mL) for 48 hours. In the culture supernatants, NO was quantified using Griess reagent, while IL-12p40 and TNF-α levels were measured by ELISA. *p < 0.05 and ***p < 0.0001, analyzed by two-way ANOVA with Bonferroni test, comparing C57Bl/6 and TLR4^-/-^ cells with the same stimulus. **(B)** BMDCs from C57Bl/6 mice were incubated as described in **(A)** and stimulated with LPS, IFN-γ, and active or heat-inactivated OpdA, with or without the TLR4 inhibitor (CLI-095, 1 µM), added 1 hour prior to stimulation. In the culture supernatants, NO was quantified using Griess reagent, while IL-12p40 and TNF-α levels were measured by ELISA. Bars represent the mean ± SD from triplicate samples. ***p < 0.0001, analyzed by one-way ANOVA with Tukey test. One of two independent experiments is shown.

For OpdA to effectively activate antigen-presenting cells, recognition is essential, so we assessed the role of TLRs. The results showed that BMDCs from TLR4^-/-^ mice significantly reduced NO and TNF-α secretion in response to OpdA or LPS stimulation. Additionally, a decrease in IL-12p40 secretion was observed, especially with heat-inactivated OpdA (**Figure 6A**). In contrast, BMDCs from TLR2^-/-^ mice did not affect cytokine and NO production (**Supplementary Figure 4A**).

Furthermore, BMDCs from C57BL/6 mice were pre-treated for one hour with CLI-095, a cyclohexane derivative that inhibits the TLR4 intracellular domain, and then exposed to either active or heat-inactivated OpdA. With this inhibitor present, the production of NO, IL-12p40, and TNF-α was completely suppressed (**Figure 4B**). Additional experiments showed that protease-activated receptors 1 and 2 (PAR-1 and PAR-2) did not participate in the activation of BMDCs by OpdA (**Supplementary Figure 4B**).

In conclusion, the findings suggest that TLR4 is involved in activating BMDCs through OpdA.

### OpdA signaling pathway involves MyD88 and TRIF

**Figure 7:**
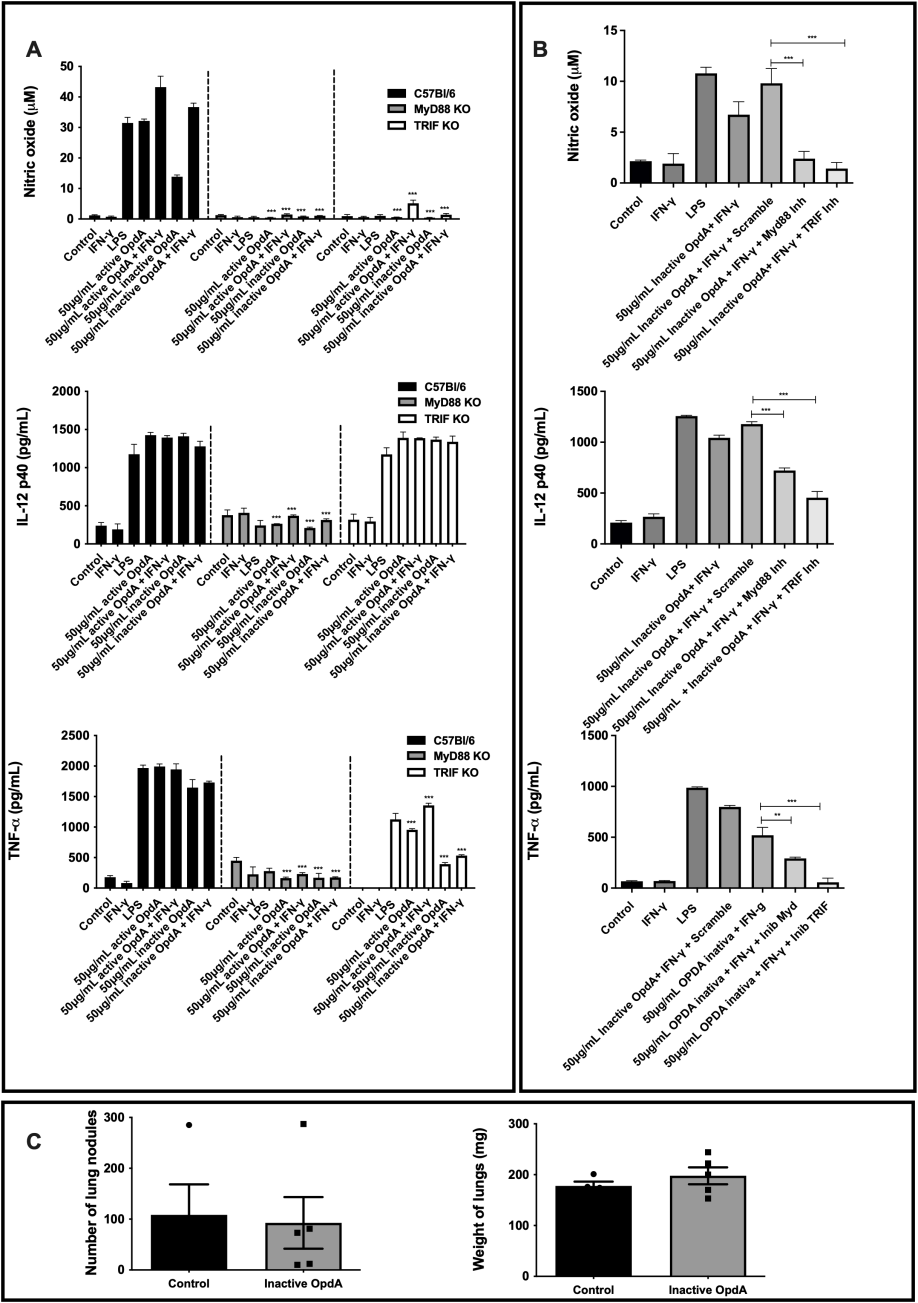
MyD88 and TRIF are necessary for the activation of BMDCs by both active and heat-inactivated OpdA. **(A)** BMDCs were derived from bone marrow progenitors of C57Bl/6, MyD88^-/-^, and TRIF^-/-^ mice, cultured in U-bottom plates (1x10^5^ cells per well), and activated with either active or heat-inactivated OpdA (50 µg/mL), LPS (200 ng/mL), and IFN-γ (200 U/mL) for 48 hours. Nitric oxide (NO) levels in the culture supernatant were measured using Griess reagent, while IL-12p40 and TNF-α were quantified via ELISA. The bars show averages ± SD from triplicate samples. Results are indicated as ***p < 0.0001, analyzed with two-way ANOVA followed by Bonferroni’s test. **(B)** BMDCs from C57Bl/6 mice were treated as in **(A)** and stimulated with LPS (200 ng/mL), IFN-γ (200 U/mL), or heat-inactivated OpdA (50 µg/mL), with or without the MyD88 inhibitor (Pepinh-MyD88, 50 µM), TRIF inhibitor (Pepinh-TRIF, 50 µM), or the control peptide (Pepinh-Control, 50 µM). NO levels in the culture supernatant were analyzed using Griess reagent, and IL-12p40 and TNF-α were measured via ELISA. The bars represent averages ± SD from triplicate samples. Results show **p < 0.001 and ***p < 0.0001, assessed by one-way ANOVA with Tukey’s test. **(C)** MyD88^-/-^ male mice (n=5) received 5x10^5^ B16F10-Nex2 melanoma cells via intravenous inoculation, followed by intraperitoneal injections of 50 µg of heat-inactivated OpdA or PBS (control) on alternate days over two weeks. Subsequently, melanotic pulmonary nodules were counted using an inverted microscope. The average lung weights are shown. This represents one of two independent experiments.

To investigate the signaling pathway responsible for the downstream activation of TLR4 by OpdA, the MyD88 and TRIF proteins were analyzed. In BMDCs from MyD88^-/-^ mice stimulated with LPS or OpdA, the production of NO, IL-12p40, and TNF-α was completely suppressed. In BMDCs from TRIF^-/-^ mice, NO production was entirely inhibited, IL-12p40 levels remained unchanged, and TNF-α showed a partial reduction (**Figure 7A**).

Additionally, BMDCs derived from C57Bl/6 mice underwent a 1-hour pre-incubation with the MyD88 inhibitory peptide (Pepinh-MyD), which disrupts MyD88 homodimerization, and the TRIF inhibitory peptide (Pepinh-TRIF), which hinders TLR-TRIF interaction, before being stimulated with heat-inactivated OpdA. As expected, the levels of NO, IL-12p40, and TNF-α were reduced due to the presence of the inhibitory peptides (**Figure 7B**).

The antitumor effect of OpdA was not observed *in vivo* in MyD88^-/-^ mice. Both treated and untreated groups had the same number of metastatic lung nodules and comparable lung weights (**Figure 7C**), confirming the *in vitro* findings.

In conclusion, the results show that MyD88 and TRIF molecules are essential for activating BMDCs by OpdA.

### The proteins in the MAPK signaling pathway play a role in the activation of BMDCs

**Figure 8:**
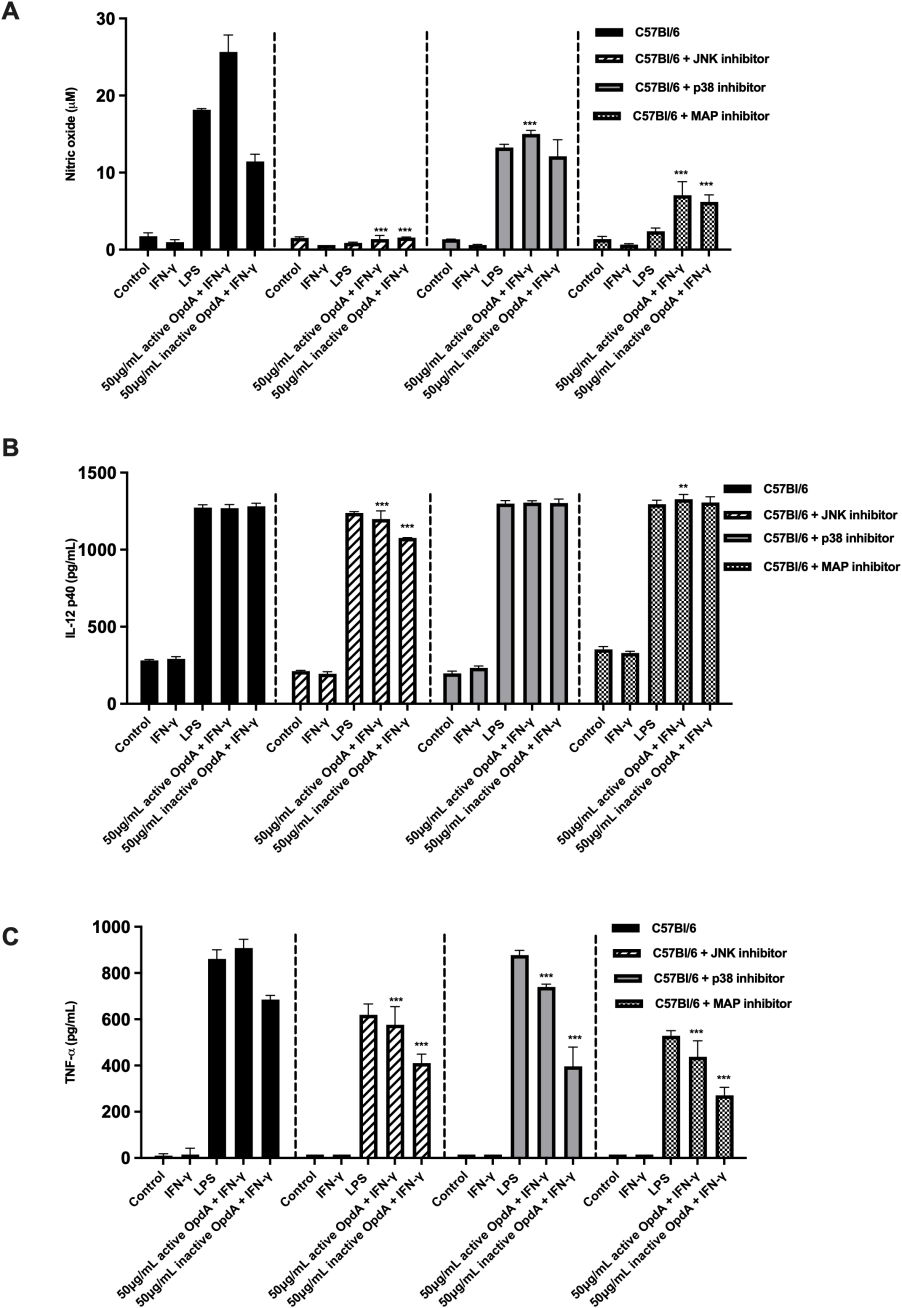
The role of proteins in the MAPK signaling pathway in activating BMDCs via active or heat-inactivated OpdA. BMDCs were obtained from C57Bl/6 mice, and 1x10^5^ cells were incubated in U-bottom plates. They were pre-treated for 1 hour with the JNK inhibitor (SP600125, 50 μM), p38 inhibitor (SB203580, 20 μM), or MAPK/ERK inhibitor (PD98059, 100 μM). Afterwards, the cells were stimulated with either active or heat-inactivated OpdA (50 μg/mL), LPS (200 ng/mL), or IFN-γ (200 U/mL) for 48 hours. The culture supernatants were then analyzed for **(A)** NO using Griess reagent; **(B)** IL-12p40; and **(C)** TNF-α via ELISA. Results are shown as averages with ± SD from triplicate samples, representing one of two independent experiments. **p < 0.001 and ***p < 0.0001, determined by two-way ANOVA with Bonferroni test, comparing data from C57Bl/6 mice without inhibitors and the same stimuli. One of two independent experiments is depicted.

The MAPK (mitogen-activated protein kinase) signaling pathways are essential for the development and function of dendritic cells. This pathway includes several proteins, such as p38, JNK (c-Jun NH2-terminal kinases), and ERK (extracellular signal-regulated protein kinases) (42, 43). To evaluate how MAPKs influence the activation of BMDCs by OpdA, the cells were pre-incubated for one hour with SP600125, SB203580, and PD98059, which serve as pharmacological inhibitors of JNK, p38, and MAP/ERK, respectively. Following this, they were stimulated with either active or heat-inactivated OpdA or LPS (used as a positive control). The production of NO stimulated by OpdA or LPS was significantly reduced by the JNK and MAP/ERK inhibitors, while the p38 inhibitor had a less pronounced impact. TNF-α production showed a partial decrease in the presence of the inhibitors, but IL-12p40 secretion remained unaffected (**Figure 8**).

These results suggest that proteins associated with the MAPK pathway contribute to the activation of BMDCs by OpdA.

## Discussion

Certain prokaryotic proteinases show immunomodulatory effects that are independent of their ability to hydrolyze proteins, although the exact mechanisms are still unclear. The heat-inactivated Sspa Subtilisin-like protease, a key virulence factor of *Streptococcus suis*, induced a dose-dependent release of IL-1β, IL-6, TNF-α, CXCL8, and CCL5 in macrophages, involving the MAPK signaling pathway (44). Arg- and Lys-gingipain cysteine proteinases, which are virulence factors of *Porphyromonas gingivalis* (the causative agent of chronic periodontitis), stimulated TNF-α and IL-8 production by monocyte-derived macrophages, even without their enzymatic activity. Furthermore, this proinflammatory response was associated with increased levels of phosphorylated p38α MAPK, indicating the pathway’s role in the process (45).

Moreover, metalloproteases from various sources have been studied as alternative therapies for tumors, showing effective results, although their mechanisms of action are still partly unclear. Bothropoidin, a eukaryotic metalloprotease from snake venom, showed *in vitro* antitumor and angiogenic effects in MDA-MB-231 breast cancer cells (46). Another eukaryotic metalloprotease, Thimed oligopeptidase (TOP), decreased the proliferation of B16F10-Nex2 melanoma murine cells *in vitro*, and only the active protease demonstrated an antitumor effect *in vivo* in mice injected subcutaneously with these tumor cells (47).

Our laboratory’s previous findings showed that the bacterial metalloprotease arazyme has an antitumor effect. The active protease significantly reduced the number of metastatic lung nodules in the B16F10-Nex2 melanoma mouse model. *In vitro*, it demonstrated a dose-dependent cytostatic effect based on its proteolytic activity, which involved decreasing CD44 expression in B16F10-Nex2 cells and reducing both the adhesion and invasion of these cells *in vitro* and *in vivo*. Additionally, arazyme stimulated the production of protease-specific IgG that can cross-react with MMP-8 from melanoma cells, thereby inhibiting the *in vivo* metastatic process (30).

We also discovered that heat-inactivated arazyme triggers an antitumor response in the B16F10-Nex2 melanoma mouse model, depending on a functioning adaptive immune system. This response is based on IFN-γ secretion and CD8^+^ T lymphocytes. Additionally, arazyme shows immunomodulatory effects by activating dendritic cells and macrophages, which increases the expression of co-stimulatory surface molecules and the release of proinflammatory cytokines. This process relies on the activation of the TLR4/MyD88/TRIF pathway, involving proteins from the MAPK signaling pathway. Furthermore, this treatment proved effective in the murine breast adenocarcinoma 4T1 model, leading to decreased primary tumor growth and metastasis, and resulting in higher survival rates in these animals (31).

We hypothesized that the antitumor and immunomodulatory effects might not be unique to bacterial arazyme. To investigate this, the current study examined the antitumor and immunomodulatory effects, along with the mechanism, of a distinct prokaryotic metalloprotease called Oligopeptidase A, found in *Salmonella typhimurium* and *Escherichia coli*. In the B16F10-Nex2 melanoma murine model, treatment with only heat-inactivated OpdA resulted in fewer lung metastatic nodules. This effect depended on the immune system, as it was ineffective in immunodeficient mice, indicating an immunomodulatory role of OpdA. A direct effect on B16F10-Nex2 tumor cells was ruled out, since neither active nor heat-inactivated OpdA affected the viability or proliferation of B16F10-Nex2 *in vitro*.

To identify the local and systemic immune effectors involved in the immunomodulatory property of OpdA, we analyzed the production of IFN-γ and IL-10 in mice treated with active and inactivated protease at the lung metastatic site, in serum, and by tumor-specific T lymphocytes in splenocyte and lymph node cell populations after *ex vivo* re-stimulation with tumor antigens.

IFN-γ is a multifunctional cytokine known for its potent antiviral, antitumor, and immunomodulatory effects. It is primarily produced by activated T lymphocytes, including NK cells, CD8^+^ T cells, and CD4^+^ T cells, as well as by antigen-presenting cells like dendritic cells and macrophages. It is one of the key cytokines produced by Th1-type T lymphocytes, which promote inflammation and fight tumor cells, while also inhibiting Th2 cell differentiation (48). In contrast, IL-10 is an immunoregulatory cytokine mainly produced by regulatory and helper T lymphocytes, as well as by tumor cells within the tumor microenvironment. Elevated levels of IL-10 are typically observed in this environment, and this cytokine is linked to poorer survival rates because it promotes tumor growth by suppressing pro-inflammatory responses, including antigen presentation, T cell proliferation, Th1 cytokine production, and cytotoxicity (49, 50). Our previous research showed that injecting a small number of B16F10Nex-2 cells into C57Bl/6 mice resulted in some animals naturally resisting tumor development. The splenocytes from these protected mice displayed high levels of IFN-γ and reduced IL-10, compared to mice that developed tumors, where immune cells produced high levels of IL-10 and lower amounts of IFN-γ. Mice genetically modified to lack IL-10 (IL-10KO) and those treated with a plasmid designed to neutralize IL-10 *in vivo*, when challenged with B16F10-Nex2 cells, exhibited enhanced survival and increased serum IFN-γ levels. However, neutralizing IFN-γ with a monoclonal antibody in these animals reversed the protective effect (51). This research emphasizes the importance of high levels of IFN-γ and low levels of IL-10, or a higher ratio of IFN-γ to IL-10, in mounting an effective protective response against murine B16F10 melanoma.

Within the pulmonary homogenate, active OpdA-treated mice showed a significant increase in the IFN-γ/IL-10 ratio. In contrast, the inactivated OpdA-treated group had a slight rise in the ratio, but this difference was not statistically significant. Only mice treated with active OpdA demonstrated an elevated IFN-γ/IL-10 ratio in serum, while none of the OpdA-treated groups showed a substantial increase in IL-10 levels.

In active OpdA-treated tumor-bearing mice, there was a significant increase in the percentages of tumor-specific CD4^+^/IFN-γ^+^ and CD4^+^/IL-10^+^ T lymphocytes in the spleen. The notable rise in CD4^+^/IFN-γ^+^ cells indicates that the IFN-γ/IL-10 ratio was higher in this group compared to others. TCD8^+^ lymphocytes showed a similar pattern, suggesting that both active and inactive OpdA-treated mice had elevated IFN-γ/IL-10 ratios. The cytokine analysis of the culture supernatant revealed an increased IFN-γ/IL-10 ratio in splenocytes and lymph node cells from active OpdA-treated mice. This highlights the important role of CD4^+^ and CD8^+^ T lymphocytes, which produce more IFN-γ than IL-10, in driving the antitumor protection provided by the metalloprotease, as demonstrated by the treatment of IFNγ^-/-^ and T cell-depleted mice with OpdA.

Furthermore, OpdA resulted in higher secretion levels of NO, IL-12p40, and TNF-α, as well as increased expression of surface CD80 and CD86 co-stimulatory molecules in BMDCs after itspex vivo/it /spactivation.

Dendritic cells (DCs) play a vital role in linking the innate and adaptive immune responses by recognizing bacterial and viral components through Toll-like receptors (TLRs) (52). The main adaptor molecules, MyD88 and TRIF, connect to the intracellular domains of TLRs. When this pathway is activated, it results in the production of cytokines such as TNF-α, IL-1β, IL-6, and IFN-α, which further activate the transcription factors NF-κB, AP-1, and IRF-3, promoting a proinflammatory response and enhancing the immune response (53). However, during tumor progression, various strong immunosuppressive mechanisms may hinder the functions of DCs and T lymphocytes, decreasing the immune system’s effectiveness against tumor cells (54). Therefore, activating TLRs with agonists is an effective strategy to boost the immune response against tumor cells (55), helping to counteract the immunosuppressive tumor environment.

Our findings showed that OpdA boosts BMDC activation through TLR4, involving MyD88 and TRIF, as demonstrated by the lack of activation in TLR4^-/-^, MyD88^-/-^, and TRIF^-/-^ mice and through specific inhibitors targeting these molecules. These results suggest that OpdA could act as a TLR4 agonist, similar to arazyme, indicating its potential as an adjuvant in antitumor vaccines.

TLRs are a group of transmembrane proteins that detect microbial and viral elements (52) and are present in various immune cells, such as DCs (55, 56).

The effectiveness of TLR4 agonists as immunoadjuvants has been thoroughly evaluated. Glucopyranosyl lipid A (GLA) effectively stimulates the *in vitro* activation of bone marrow-derived dendritic cells (BMDCs) through TLR4. Moreover, in a study using DC-targeted lentiviral vectors and GLA, strong antitumor effects were seen alongside increased CD8^+^ and CD4^+^ T cell responses that depended on MyD88 (57). The lipid A TLR4 agonist improved T cell survival and proliferation in a T cell priming adoptive transfer model and promoted dendritic cell maturation. These results depended on TRIF, with type I interferon production playing a crucial role in this adjuvant effect (58). Additionally, λ-carrageenan (λ-CGN) activated DCs and boosted cytokine secretion via TLR4. In the TC-1 tumor mouse model, treatment with λ-CGN-pulsed DCs and HPV peptides effectively slowed tumor growth and triggered a strong CD8^+^ T cell response (59). The bacterial derivative Poly (γ-glutamic acid) (γ-PGA) also stimulated BMDC activation and cross-priming through TLR4 stimulation. Mice immunized with γ-PGA and later challenged with EG7-OVA tumor cells showed significant tumor growth reduction, better survival, and strong memory responses (60). In the B16 melanoma mouse model, immunization with lumazine synthase from *Brucella abortus* (BSL) led to tumor growth suppression and increased survival mediated by TLR4 (61).

Since OpdA was derived from recombinant bacteria, a key concern was the potential presence of endotoxin contaminants in the samples, which could stimulate TLR4 and cause the observed antitumor and immunomodulatory effects. To rule this out, we performed experiments with polymyxin B, which inhibits the biological effects of LPS, and treated OpdA with proteinase K, which selectively cleaves OpdA without affecting LPS. In the polymyxin B experiments, we observed a partial reduction in BMDC activation, suggesting low-dose endotoxin presence in the sample. However, when OpdA was degraded with proteinase K, BMDC stimulation was not observed, indicating that the main effect is from OpdA rather than contaminants. Additionally, activating BMDCs with LPS at concentrations equivalent to the endotoxin levels measured in the OpdA preparation (via the LAL assay) was ineffective, and no synergistic effect between OpdA and LPS was detected.

The involvement of proteins from the MAPK signaling pathway in producing IL-12p40, TNF-α, and NO by OpdA-activated BMDCs was also demonstrated. Using specific inhibitors for JNK, p38, and MAPK, it was confirmed that OpdA activates BMDCs through this pathway. The MAPK cascade is essential for various cellular functions, including the proliferation and differentiation of immune cells such as dendritic cells, as well as the regulation and expression of proinflammatory cytokines like IL-12, TNF-α, IL-1β, and IL-6 (42, 62, 63). Other TLR4 ligands also help activate the MAPK cascade. For example, serum amyloid A (SAA) stimulated NO production in peripheral macrophages by activating ERK1/2 and p38 (64). Additionally, the *Ganoderma atrum* polysaccharide (PSG-1) activated macrophages and increased the production of TNF-α, IL-1β, and NO via p38 MAPK in the murine CT26 tumor model (65). Other bacterial proteases, such as gingipain (45, 66) and subtilisin-like protease (44), are also linked to MAPK cascade activation.

We also investigated the role of additional receptors in activating BMDCs by OpdA. Protease-activated receptors (PARs), a type of G-protein-coupled receptor, are found in nearly all cell types, including dendritic cells (67). These receptors can trigger innate immunity and initiate inflammatory responses. Typically, PAR activation occurs when proteases cleave the receptor; for example, thrombin activates PAR-1 (68). This cleavage exposes the N-terminus, causing structural changes and starting downstream signaling pathways (69). Maharshak et al. (70) recently showed that gelatinase from *Enterococcus faecalis* can increase enteric permeability, leading to intestinal inflammation via PAR-2. Our results indicated that blocking PAR-1 and PAR-2 with antagonistic peptides did not affect the stimulation of BMDCs by active OpdA. This suggests that these receptors are not activated by the bacterial protease OpdA. Additionally, TLR2 did not influence BMDC activation by OpdA, as demonstrated by the lack of effect in TLR2^-/-^ mice.

Recent findings highlight a significant impact of immunotherapies on skeletal muscles. Immune checkpoint inhibitors have been linked to immune-related adverse effects on the musculoskeletal system, while adoptive cell immunotherapy can induce cytokine release syndrome, disrupting muscle cell metabolism and function. Tumor vaccines may cause symptoms like muscle fatigue, soreness, and discomfort in some individuals. Changes in the tumor microenvironment also affect skeletal muscle metabolism and function through multiple pathways. Clinical data indicate that about 30–50% of cancer patients experience notable declines in skeletal muscle function after chemotherapy, with higher rates among the elderly and those in poor health. This decline mainly involves muscle mass reduction, primarily due to the direct toxic effects of immunotherapy on muscle tissue, unrelated to tumor response. Loss of muscle mass significantly impairs physical mobility and daily life quality, and may also decrease chemotherapy tolerance, increase drug toxicity, and negatively influence overall survival. Similar effects are observed with chemotherapeutic drugs, raising concerns, especially as combined chemotherapy and immunotherapy become more common in practice (71, 72).

Our current work has not examined the effect of OpdA treatment on mouse muscle cells *in vivo* or its impact on human immune and tumor cells. Future studies should address these questions.

In conclusion, our findings show that OpdA can trigger an antitumor response in the B16F10-Nex2 melanoma mouse model, regardless of its enzymatic activity. An immunomodulatory effect on antigen-presenting cells (APCs) was observed, involving TLR4/MyD88/TRIF and members of the MAPK signaling pathway. These results suggest that OpdA could serve as an adjuvant in tumor vaccines, enhancing the antigen-presenting process and strengthening the tumor-specific immune response. Furthermore, the immunomodulatory effect identified may extend beyond arazyme and OpdA, possibly indicating that metalloproteinases from other sources could also have immunomodulatory effects, which future research should explore.

## Data Availability

The raw data supporting the conclusions of this article will be made available by the authors, without undue reservation.

## Supplementary material

Supplementary Figure 1 Purification and enzymatic activity of recombinant OpdA (rOpdA). **(A)** Purified rOpdA was analyzed using 10% SDS-PAGE followed by silver staining. Lane 1: molecular weight marker; Lanes 2–4: 5 μg, 10 μg, and 15 μg of heat-inactivated rOpdA, respectively. In Lane 5, 5 μg of heat-inactivated rOpdA was subjected to Western blot analysis using a mouse-derived anti-OpdA primary antibody and an alkaline phosphatase-conjugated anti-mouse IgG secondary antibody. **(B)** The structural integrity of rOpdA under reducing and non-reducing conditions was assessed. Lane 1: molecular weight marker; Lanes 2–4: 1 μg, 2 μg, and 5 μg of heat-inactivated rOpdA, respectively; Lanes 5–7: 1 μg, 2 μg, and 5 μg of active rOpdA, respectively. **(C)** Proteolytic degradation of rOpdA was performed using proteinase K. Lane 1: molecular weight marker; Lane 2: 5 μg of rOpdA; Lanes 3 and 4: 5 μg of rOpdA degraded with proteinase K. For enzymatic activity analysis, purified rOpdA was incubated with the fluorogenic substrate Abz-GFSIFRQ–EDDnp (20 μM) in 50 mM Tris-HCl buffer (pH 7.4) at 37 °C. Hydrolysis of the fluorogenic substrate was monitored by fluorescence measurement at λem=420 nm and λex=320 nm. **(D)** The enzymatic activity of both active and heat-inactivated rOpdA was evaluated, along with the **(E)** kinetic curve of the active form.

Supplementary Figure 2 The immune system’s role in the *in vivo* antitumor activity of heat-inactive rOpdA. **(A)** C57Bl/6 male mice and **(B)** NSG mice were injected intravenously with 5x10^5^ B16F10-Nex2 melanoma cells, followed by intraperitoneal treatment with 50µg of either active or heat-inactive rOpdA, or PBS (control), every other day for two weeks. After treatment, the number of melanotic pulmonary nodules was counted using an inverted microscope. The figure shows representative lung images from each group, **(A)** C57Bl/6 mice and **(B)** NSG mice.

Supplementary Figure 3 Tumor cell proliferation assay with rOpdA. B16F10-Nex2 melanoma murine cells were treated with different concentrations of active rOpdA **(A)** or heat-inactive rOpdA **(B)** for 24 or 48 hours. The data show the mean ± SD from triplicate samples. Each concentration was compared to the negative control (0 μg/mL). ** p < 0.001, and *** p < 0.0001, analyzed by one-way ANOVA with Tukey’s test.

Supplementary Figure 4 APC activation by active or inactive rOpdA does not depend on TLR2, PAR-1, or PAR-2. **(A)** APCs from TLR2^-/-^ and C57Bl/6 mice were incubated in U-bottom plates (1x10^5^ cells/well) and then stimulated with active or heat-inactivated rOpdA (50 µg/mL), LPS (200 ng/mL), and IFN-γ (200 U/mL) for 48 hours. NO was measured in the culture supernatants using Griess reagent, and IL-12 (p40) and TNF-α levels were determined by ELISA. **(B)** APCs from C57Bl/6 mice were incubated as described and pretreated for 1 hour with the reverse peptides of PAR-1 (5 or 10 µM) or PAR-2 (20 or 40 µM). Subsequently, these cells were stimulated with LPS (200 ng/mL), IFN-γ (200 U/mL), and active rOpdA (50 µg/mL). The secretion of NO, IL-12(p40), and TNF-α was analyzed by ELISA. Results are shown as the average ± SD from triplicate samples. Data from one of two independent experiments are presented. *** p < 0.0001, analyzed by two-way ANOVA with Bonferroni post-test, comparing C57Bl/6 mice with the same stimulus.

## References

[1] Moreira J Moreira E Azevedo F Mota A. Cutaneous Malignant melanoma: a retrospective study of seven years. Acta Med Port. (2014) 27:480–8. doi: 10.20344/amp.5211 25203957

[2] Lodde G Albrecht LJ SChadendorf D. Treatment of metastatic melanoma: update 2025. Dtsch Med Wochenschr. (2025) 150:562–9. doi: 10.1055/a-2500-0927, PMID: 40262755

[3] Jiyao Y Fu L Wu R Che L Liu G Ran Q. Immunocytes in the tumor microenvironment: recent updates and interconnections. Front Immunol. (2025) 16:1517959. doi: 10.3389/fimmu.2025.1517959, PMID: 40297580 PMC12034658

[4] Tomar S Siddiqui S Pathak R Vivek S. Unlocking the potential of immunomodulators as synergistic immune-based therapies in cancer. Discov Med. (2025) 37:411–32. doi: 10.24976/Discov.Med.202537194.35, PMID: 40116091

[5] Coffman RL Sher A Seder RA. Vaccine adjuvants: putting innate immunity to work. Immunity. (2010) 33:492–503. doi: 10.1016/j.immuni.2010.10.002, PMID: 21029960 PMC3420356

[6] Temizoz B Kuroda E Ishii KJ. Vaccine adjuvants as potential cancer immunotherapeutics. Int Immunol. (2016) 28:329–38. doi: 10.1093/intimm/dxw015, PMID: 27006304 PMC4922024

[7] Ma Y Shurin GV Gutkin DW Shurin MR. Tumor-associated regulatory dendritic cells. Semin Cancer Biol. (2012) 22:298–306. doi: 10.1016/j.semcancer.2012.02.010, PMID: 22414911 PMC3373995

[8] Constantino J Gomes C Falcao A Neves BM Cruz MT. Dendritic cell-based immunotherapy: a basic review and recent advances. Immunol Res. (2017) 65:798–810. doi: 10.1007/s12026-017-8931-1, PMID: 28660480

[9] Garg AD Vara Perez M Schaaf M Agostinis P Zitvogel L Kroemer G. Trial watch: Dendritic cell-based anticancer immunotherapy. Oncoimmunology. (2017) 6:e1328341. doi: 10.1080/2162402X.2017.1328341, PMID: 28811970 PMC5543823

[10] Ardavin C Amigorena S Reis e Sousa C. Dendritic cells: immunobiology and cancer immunotherapy. Immunity. (2004) 20:17–23. doi: 10.1016/s1074-7613(03)00352-2, PMID: 14738761

[11] Palucka K Banchereau J. Cancer immunotherapy via dendritic cells. Nat Rev Cancer. (2012) 12:265–77. doi: 10.1038/nrc3258, PMID: 22437871 PMC3433802

[12] Bol KF Figdor CG Aarntzen EH Welzen ME van Rossum MM Blokx WA. Intranodal vaccination with mRNA-optimized dendritic cells in metastatic melanoma patients. Oncoimmunology. (2015) 4:e1019197. doi: 10.1080/2162402X.2015.1019197, PMID: 26405571 PMC4570143

[13] Van Brussel I Berneman ZN Cools N. Optimizing dendritic cell-based immunotherapy: tackling the complexity of different arms of the immune system. Mediators Inflamm. (2012) 2012:690643. doi: 10.1155/2012/690643, PMID: 22851815 PMC3407661

[14] Leroux-Roels G. Unmet needs in modern vaccinology: adjuvants to improve the immune response. Vaccine. (2010) 28:C25–36. doi: 10.1016/j.vaccine.2010.07.021, PMID: 20713254

[15] Thomas S Prendergast GC. Cancer Vaccines: A brief overview. Methods Mol Biol. (2016) 1403:755–61. doi: 10.1007/978-1-4939-3387-7 27076165

[16] Chang ZL. Essential aspects of Toll-like receptors, ligands, and their signaling pathways. Inflammation Res. (2010) 59:791–808. doi: 10.1007/s00011-010-0208-2, PMID: 20593217

[17] Takeda K Akira S. Toll-like receptors. Curr Protoc Immunol. (2015) 109. doi: 10.1002/0471142735.im1412s109, PMID: 25845562

[18] Plociennikowska A Hromada-Judycka A Borzecka K Kwiatkowska K. Co-operation of TLR4 and raft proteins in LPS-induced pro-inflammatory signaling. Cell Mol Life Sci. (2015) 72:557–81. doi: 10.1007/s00018-014-1762-5, PMID: 25332099 PMC4293489

[19] Guha M Mackman N. LPS induction of gene expression in human monocytes. Cell Signal. (2001) 13:85–94. doi: 10.1016/S0898-6568(00)00149-2, PMID: 11257452

[20] Fichter M Dedters M Pietrzak-Nguyen A Pretsch L Meyer CU Strand S. Monophosphoryl lipid A coating of hydroxyethyl starch nanocapsules drastically increases uptake and maturation by dendritic cells while minimizing the adjuvant dosage. Vaccine. (2015) 33:838–46. doi: 10.1016/j.vaccine.2014.12.072, PMID: 25573037

[21] Mitchell MS Harel W Kempf RA Hu E Kan-Mitchell J Boswell WD. Active-specific immunotherapy for melanoma. J Clin Oncol. (1990) 8:856–69. doi: 10.1200/JCO.1990.8.5.856, PMID: 2139701

[22] Bhatla N Suri V Basu P Shastri S Datta SK Bi D. Immunogenicity and safety of human papillomavirus-16/18 AS04-adjuvanted cervical cancer vaccine in healthy Indian women. J Obstet Gynaecol Res. (2010) 36:123–32. doi: 10.1111/j.1447-0756.2009.01167.x, PMID: 20178538

[23] Engström KK Khalaf H Kälvegren H Bengtsson T. The role of Porphyromonas gingivalis gingipains in platelet activation and innate immune modulation. Mol Oral Microbiol. (2015) 30:62–73. doi: 10.1111/omi.12067, PMID: 25043711

[24] Glowczyk I Wong A Potempa B Babyak O Lech M Lamont RJ. Inactive gingipains from P. gingivalis selectively skew T cells toward a Th17 phenotype in an IL-6 dependent manner. Front Cell Infect Microbiol. (2017) 7:140. doi: 10.3389/fcimb.2017.00140, PMID: 28497028 PMC5406403

[25] Bellelli A Mattioni M Rusconi V Sezzi ML Bellelli L. Inhibition of tumor growth, invasion, and metastasis in papain-immunized mice. Invasion Metastasis. (1990) 10:142–69., PMID: 2139872

[26] Eckert K Grabowska E Stange R Schneider U Eschmann K Maurer HR. Effects of oral bromelain administration on the impaired immunocytotoxicity of mononuclear cells from mammary tumor patients. Oncol Rep. (1999) 6:1191–9. doi: 10.3892/or.6.6.1191, PMID: 10523679

[27] Maurer HR. Bromelain: biochemistry, pharmacology and medical use. Cell Mol Life Sci. (2001) 58:1234–45. doi: 10.1007/PL00000936, PMID: 11577981 PMC11337410

[28] Guimaraes-Ferreira CA Rodrigues EG Mortara RA Cabral H Serrano FA Ribeiro-dos-Santos R. Antitumor effects *in vitro* and *in vivo* and mechanisms of protection against melanoma B16F10-Nex2 cells by fastuosain, a cysteine proteinase from Bromelia fastuosa. Neoplasia. (2007) 9:723–33. doi: 10.1593/neo.07427, PMID: 17898868 PMC1993857

[29] Bersanetti PA Park H Bae KS Son K Shin D Hirata IY. Characterization of arazyme, an exocellular metalloprotease isolated from Serratia proteamaculans culture medium. Enzyme Microb Technol. (2005) 34:574–81. doi: 10.1016/j.enzmictec.2005.01.041

[30] Pereira FV Ferreira-Guimaraes CA Paschoalin T Scutti JA Melo FM Silva LS. A natural bacterial-derived product, the metalloprotease arazyme, inhibits metastatic murine melanoma by inducing MMP-8 cross-reactive antibodies. PloS One. (2014) 9:e96141. doi: 10.1371/journal.pone.0096141, PMID: 24788523 PMC4005744

[31] Pereira FV Melo AC de Melo FM Mourao-Sa D Silva P Berzaghi R. TLR4-mediated immunomodulatory properties of the bacterial metalloprotease arazyme in preclinical tumor models. Oncoimmunology. (2016) 5:e1178420. doi: 10.1080/2162402X.2016.1178420, PMID: 27622031 PMC5006932

[32] Paschoalin T Carmona AK Oliveira V Juliano L Travassos LR. Characterization of thimet- and neurolysin-like activities in Escherichia coli M 3 A peptidases and description of a specific substrate. Arch Biochem Biophys. (2005) 441:25–34. doi: 10.1016/j.abb.2005.06.011, PMID: 16098472

[33] Jain R Chan MK. Support for a potential role of E. coli oligopeptidase A in protein degradation. Biochem Biophys Res Commun. (2007) 359:486–90. doi: 10.1016/j.bbrc.2007.05.142, PMID: 17553460

[34] Lorenzon RZ Cunha CE Marcondes MF MaChado MF Juliano MA Oliveira V. Kinetic characterization of the Escherichia coli oligopeptidase A (OpdA) and the role of the Tyr(607) residue. Arch Biochem Biophys. (2010) 500:131–6. doi: 10.1016/j.abb.2010.05.025, PMID: 20513640

[35] Fidler IJ. Biological behavior of Malignant melanoma cells correlated with their survival *in vivo* . Cancer Res. (1975) 35:218–24., PMID: 1109790

[36] Freitas ZF Rodrigues EG Oliveira V Carmona AK Travassos LR. Melanoma heterogeneity: differential, invasive, metastatic properties and profiles of cathepsin B, D, and L activities in subclones of the B16F10-NEX2 cell line. Melanoma Res. (2004) 14:333–44. doi: 10.1097/00008390-200410000-00002, PMID: 15457088

[37] Dobroff AS Rodrigues EG Moraes JZ Travassos LR. Protective, anti-tumor monoclonal antibody recognizes a conformational epitope similar to melibiose at the surface of invasive murine melanoma cells. Hybrid Hybridomics. (2002) 21:321–31. doi: 10.1089/153685902761022661, PMID: 12470474

[38] Dias BR Rodrigues EG Nimrichter L Nakayasu E Almeida IC Travassos LR. Identification of iGb3 and iGb4 in melanoma B16F10-Nex2 cells and the iNKT cell-mediated antitumor effect of dendritic cells primed with iGb3. Mol Cancer. (2009) 8:116. doi: 10.1186/1476-4598-8-116, PMID: 19968878 PMC2795753

[39] Green LC Wagner DA Glogowski J Skipper PL Wishnok JS Tannenbaum SR. Analysis of nitrate, nitrite, and [15N]nitrate in biological fluids. Anal Biochem. (1982) 126:131–8. doi: 10.1016/0003-2697(82)90118-X, PMID: 7181105

[40] Tynan GA McNaughton A Jarnicki A Tsuji T Lavelle EC. Polymyxin B inadequately quenches the effects of contaminating lipopolysaccharide on murine dendritic cells. PloS One. (2012) 7:e37261. doi: 10.1371/journal.pone.0037261, PMID: 22624003 PMC3356265

[41] Ebeling W Hennrich N Klockow M Metz H Orth HD Lang H. Proteinase K from tritirachium album limber. Eur J Biochem. (1974) 47:91–7. doi: 10.1111/j.1432-1033.1974.tb03671.x, PMID: 4373242

[42] Dong C Davis RJ Flavell RA. MAP kinases in the immune response. Annu Rev Immunol. (2002) 20:55–72. doi: 10.1146/annurev.immunol.20.091301.131133, PMID: 11861597

[43] Dáňová K Klapetková A Kayserová J Šedivá A Špíšek R Jelínková LP. NF-κB, p38 MAPK, ERK1/2, mTOR, STAT3 and increased glycolysis regulate stability of paricalcitol/dexamethasone-generated tolerogenic dendritic cells in the inflammatory environment. Oncotarget. (2015) 6:14123–38. doi: 10.18632/oncotarget.4234, PMID: 26053099 PMC4546455

[44] Bonifait L Grenier D. The SspA subtilisin-like protease of Streptococcus suis triggers a pro-inflammatory response in macrophages through a non-proteolytic mechanism. BMC Microbiol. (2011) 11:47. doi: 10.1186/1471-2180-11-47, PMID: 21362190 PMC3058005

[45] Grenier D Tanabe S. Porphyromonas gingivalis gingipains trigger a proinflammatory response in human monocyte-derived macrophages through the p38alpha mitogen-activated protein kinase signal transduction pathway. Toxins (Basel). (2010) 2:341–52. doi: 10.3390/toxins2030341, PMID: 22069588 PMC3153194

[46] Guimaraes DO Lopes DS Azevedo FV Gimenes SN Silva MA Ache DC. *In vitro* antitumor and antiangiogenic effects of Bothropoidin, a metalloproteinase from Bothrops pauloensis snake venom. Int J Biol Macromol. (2017) 97:770–7. doi: 10.1016/j.ijbiomac.2017.01.064, PMID: 28093334

[47] Paschoalin T Carmona AK Rodrigues EG Oliveira V Monteiro HP Juliano MA. Characterization of thimet oligopeptidase and neurolysin activities in B16F10-Nex2 tumor cells and their involvement in angiogenesis and tumor growth. Mol Cancer. (2007) 6:44. doi: 10.1186/1476-4598-6-44, PMID: 17620116 PMC1965469

[48] Castro F Cardoso AP Gonçalves RM Serre K Oliveira MJ. Interferon-gamma at the crossroads of tumor immune surveillance or evasion. Front Immunol. (2018) 9:847. doi: 10.3389/fimmu.2018.00847, PMID: 29780381 PMC5945880

[49] Itakura E Huang RR Wen DR Paul E Wünsch PH Cochran AJ. IL10 expression by primary tumor cells correlates with melanoma progression from radial to vertical growth phase and development of metastatic competence. Mod Pathol. (2011) 24:801–9. doi: 10.1038/modpathol.2011.5, PMID: 21317876 PMC3106125

[50] Zhao S Wu D Wu P Wang Z Huang J. Serum IL10 predicts worse outcome in cancer patients: a meta-analysis. PloS One. (2015) 10:e0139598. doi: 10.1371/journal.pone.0139598, PMID: 26440936 PMC4595202

[51] Marchi LH Paschoalin T Travassos LR Rodrigues EG. Gene therapy with interleukin-10 receptor and interleukin-12 induces a protective interferon-γ-dependent response against B16F10-Nex2 melanoma. Cancer Gene Ther. (2011) 18:110–22. doi: 10.1038/cgt.2010.58, PMID: 20885448

[52] Toussi DN Massari P. Immune adjuvant effect of molecularly-defined Toll-Like Receptor ligands. Vaccines (Basel). (2014) 2:323–53. doi: 10.3390/vaccines2020323, PMID: 26344622 PMC4494261

[53] Amos SM Pegram HJ Westwood JA John LB Devaud C Clarke CJ. Adoptive immunotherapy combined with intratumoral TLR agonist delivery eradicates established melanoma in mice. Cancer Immunol Immunother. (2011) 60:671–83. doi: 10.1007/s00262-011-0984-8, PMID: 21327636 PMC3499620

[54] Mellman I. Dendritic cells: master regulators of the immune response. Cancer Immunol Res. (2013) 1:145–9. doi: 10.1158/2326-6066.CIR-13-0102, PMID: 24777676

[55] Shi M Chen X Ye K Yao Y Li Y. Application potential of toll-like receptors in cancer immunotherapy: Systematic review. Med (Baltimore). (2016) 95:e3951. doi: 10.1097/MD.0000000000003951, PMID: 27336891 PMC4998329

[56] Smith SG Zaharoff DA. Future directions in bladder cancer immunotherapy: towards adaptive immunity. Immunotherapy. (2016) 8:351–65. doi: 10.2217/imt.15.122, PMID: 26860539 PMC5618954

[57] Xiao L Kim J Lim M Dai B Yang L Reed SG. A TLR4 agonist synergizes with dendritic cell-directed lentiviral vectors for inducing antigen-specific immune responses. Vaccine. (2012) 30:2570–81. doi: 10.1016/j.vaccine.2012.01.074, PMID: 22314134 PMC3360926

[58] Gandhapudi SK Chilton PM Mitchell TC. TRIF is required for TLR4-mediated adjuvant effects on T cell clonal expansion. PloS One. (2013) 8:e56855. doi: 10.1371/journal.pone.0056855, PMID: 23457630 PMC3574014

[59] Li J Aipire A Zhu H Wang Y Guo W Li X. lambda-Carrageenan improves the antitumor effect of dendritic cell-based vaccine. Oncotarget. (2017) 8:29996–30007. doi: 10.18632/oncotarget.15610, PMID: 28404904 PMC5444720

[60] Seth A Heo MB Sung MH Lim YT. Infection-mimicking poly(gamma-glutamic acid) as an adjuvant material for effective anti-tumor immune response. Int J Biol Macromol. (2015) 75:495–504. doi: 10.1016/j.ijbiomac.2015.02.013, PMID: 25709015

[61] Rossi AH Farias A Fernandez JE Bonomi HR Goldbaum FA Berguer PM. Brucella spp. Lumazine Synthase induces a TLR4-Mediated protective response against B16 Melanoma in Mice. PloS One. (2015) 10:e0126827. doi: 10.1371/journal.pone.0126827, PMID: 25973756 PMC4431812

[62] Wang X Liu Y. Regulation of innate immune response by MAP kinase phosphatase-1. Cell Signal. (2007) 19:1372–82. doi: 10.1016/j.cellsig.2007.03.013, PMID: 17512700 PMC2203964

[63] Low HB Zhang Y. Regulatory roles of MAPK phosphatases in cancer. Immune Netw. (2016) 16:85–98. doi: 10.4110/in.2016.16.2.85, PMID: 27162525 PMC4853501

[64] Sandri S Rodriguez D Gomes E Monteiro HP Russo M Campa A. Is serum amyloid A an endogenous TLR4 agonist? J Leukoc Biol. (2008) 83:1174–80. doi: 10.1189/jlb.0407203, PMID: 18252871

[65] Zhang S Nie S Huang D Li W Xie M. Immunomodulatory effect of Ganoderma atrum polysaccharide on CT26 tumor-bearing mice. Food Chem. (2013) 136:1213–9. doi: 10.1016/j.foodchem.2012.08.090, PMID: 23194516

[66] Fitzpatrick RE Aprico A Wijeyewickrema LC Pagel CN Wong DM Potempa J. High molecular weight gingipains from Porphyromonas gingivalis induce cytokine responses from human macrophage-like cells via a nonproteolytic mechanism. J Innate Immun. (2009) 1:109–17. doi: 10.1159/000181145, PMID: 20375569 PMC3087433

[67] Zhang H Wang M Shi T Shen L Zhu J Sun M. Genetic polymorphisms of PAI-1 and PAR-1 are associated with acute normal tissue toxicity in Chinese rectal cancer patients treated with pelvic radiotherapy. Onco Targets Ther. (2015) 8:2291–301. doi: 10.2147/OTT.S83723, PMID: 26347502 PMC4556037

[68] Grimsey NJ Trejo J. Integration of endothelial protease-activated receptor-1 inflammatory signaling by ubiquitin. Curr Opin Hematol. (2016) 23:274–9. doi: 10.1097/MOH.0000000000000232, PMID: 26845544 PMC4978167

[69] Zhao P Metcalf M Bunnett NW. Biased signaling of protease-activated receptors. Front Endocrinol (Lausanne). (2014) 5:67. doi: 10.3389/fendo.2014.00067, PMID: 24860547 PMC4026716

[70] Maharshak N Huh EY Paiboonrungruang C Shanahan M Thurlow L Herzog J. Enterococcus faecalis Gelatinase mediates intestinal permeability via Protease-Activated Receptor 2. Infect Immun. (2015) 83:2762–70. doi: 10.1128/IAI.00425-15, PMID: 25916983 PMC4468563

[71] Ma S Zhao G Sui S Chen X Wu L Wang T. Tumor microenvironment and immune-related myositis: addressing muscle wasting in cancer immunotherapy. Front Immunol. (2025) 16:1580108. doi: 10.3389/fimmu.2025.1580108, PMID: 40386783 PMC12081358

[72] Ma S Lu Y Sui S Yang JS Fu BB Tan PX. Unraveling the triad of immunotherapy, tumor microenvironment, and skeletal muscle biomechanics in oncology. Front Immunol. (2025) 16:1572821. doi: 10.3389/fimmu.2025.1572821, PMID: 40242775 PMC12000078

