# Supplementary figures and images for: The immunomodulatory and antitumor properties of the bacterial metalloprotease Oligopeptidase A are mediated by TLR4/MyD88/TRIF and MAPK signaling pathways
Source: Front Immunol. 2025 Sep 12;16:1630886. doi: 10.3389/fimmu.2025.1630886 (PMC12463843; doi:10.3389/fimmu.2025.1630886)

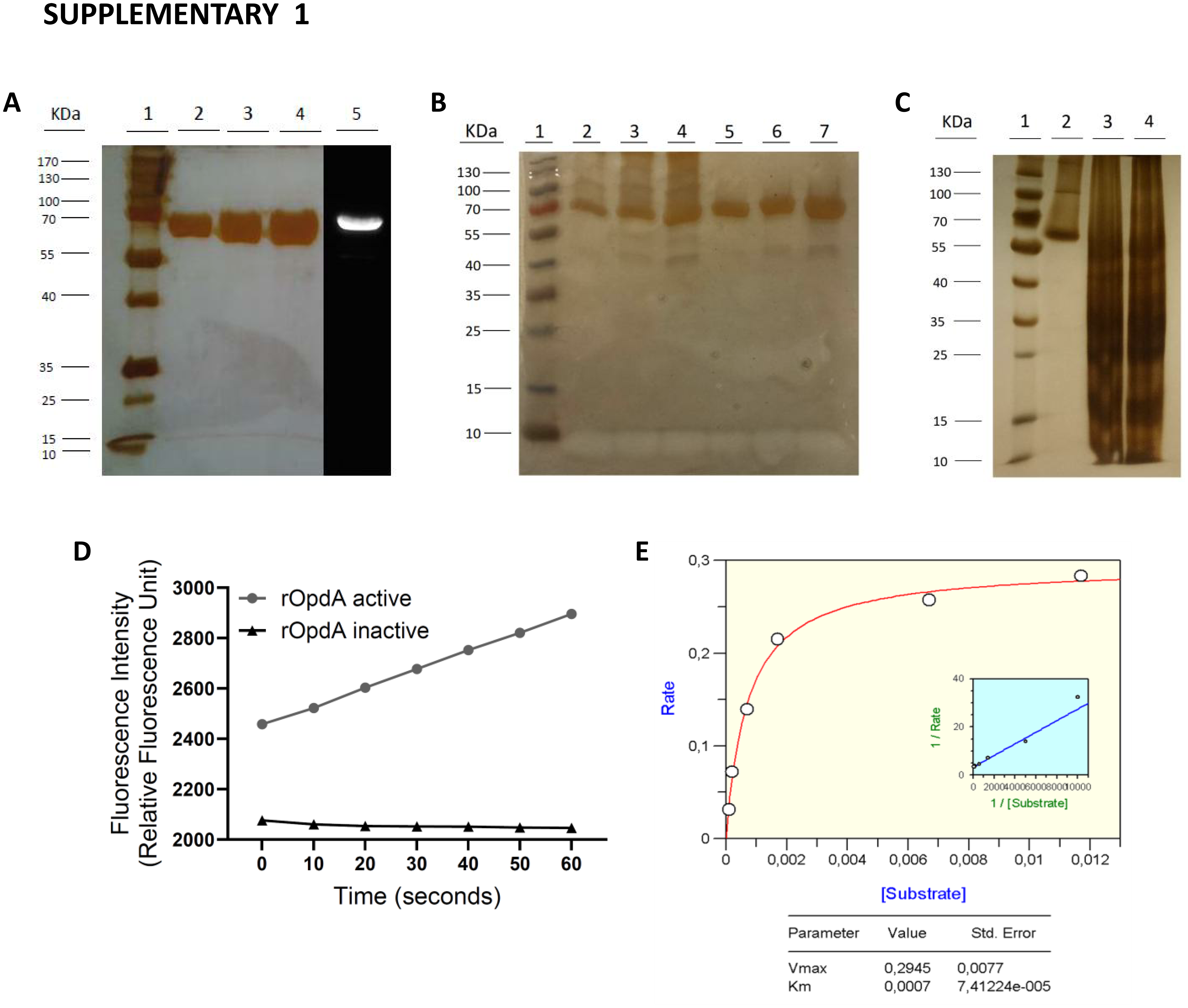

Supplement: Supplementary Figure 1 — Purification and enzymatic activity of recombinant OpdA (rOpdA). (A) Purified rOpdA was analyzed using 10% SDS-PAGE followed by silver staining. Lane 1: molecular weight marker; Lanes 2–4: 5 μg, 10 μg, and 15 μg of heat-inactivated rOpdA, respectively. In Lane 5, 5 μg of heat-inactivated rOpdA was subjected to Western blot analysis using a mouse-derived anti-OpdA primary antibody and an alkaline phosphatase-conjugated anti-mouse IgG secondary antibody. (B) The structural integrity of rOpdA under reducing and non-reducing conditions was assessed. Lane 1: molecular weight marker; Lanes 2–4: 1 μg, 2 μg, and 5 μg of heat-inactivated rOpdA, respectively; Lanes 5–7: 1 μg, 2 μg, and 5 μg of active rOpdA, respectively. (C) Proteolytic degradation of rOpdA was performed using proteinase K. Lane 1: molecular weight marker; Lane 2: 5 μg of rOpdA; Lanes 3 and 4: 5 μg of rOpdA degraded with proteinase K. For enzymatic activity analysis, purified rOpdA was incubated with the fluorogenic substrate Abz-GFSIFRQ–EDDnp (20 μM) in 50 mM Tris-HCl buffer (pH 7.4) at 37 °C. Hydrolysis of the fluorogenic substrate was monitored by fluorescence measurement at λem=420 nm and λex=320 nm. (D) The enzymatic activity of both active and heat-inactivated rOpdA was evaluated, along with the (E) kinetic curve of the active form. [file Image1.tif]

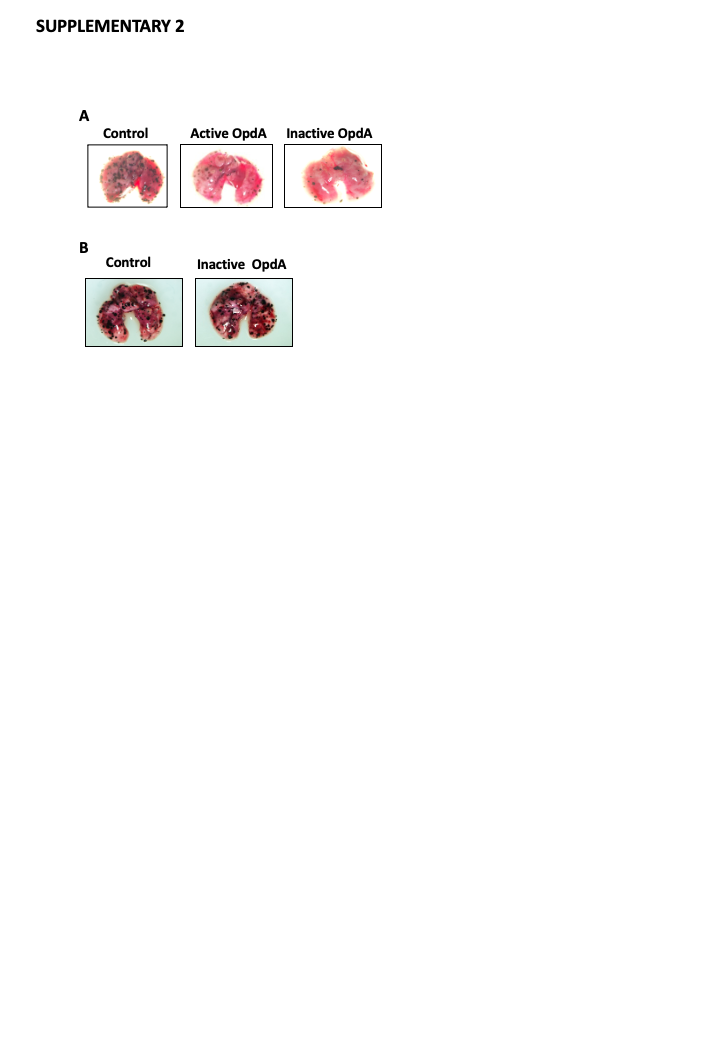

Supplement: Supplementary Figure 2 — The immune system’s role in the in vivo antitumor activity of heat-inactive rOpdA. (A) C57Bl/6 male mice and (B) NSG mice were injected intravenously with 5x10^5^ B16F10-Nex2 melanoma cells, followed by intraperitoneal treatment with 50µg of either active or heat-inactive rOpdA, or PBS (control), every other day for two weeks. After treatment, the number of melanotic pulmonary nodules was counted using an inverted microscope. The figure shows representative lung images from each group, (A) C57Bl/6 mice and (B) NSG mice. [file Image2.tiff]

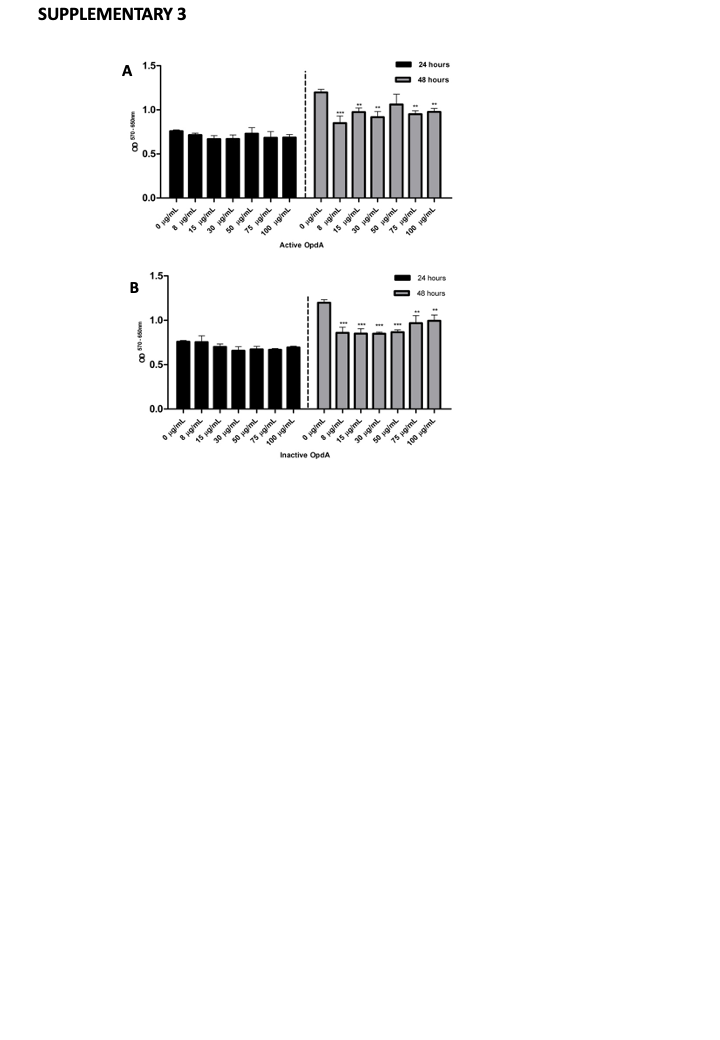

Supplement: Supplementary Figure 3 — Tumor cell proliferation assay with rOpdA. B16F10-Nex2 melanoma murine cells were treated with different concentrations of active rOpdA (A) or heat-inactive rOpdA (B) for 24 or 48 hours. The data show the mean ± SD from triplicate samples. Each concentration was compared to the negative control (0 μg/mL). ** p < 0.001, and *** p < 0.0001, analyzed by one-way ANOVA with Tukey’s test. [file Image3.tiff]

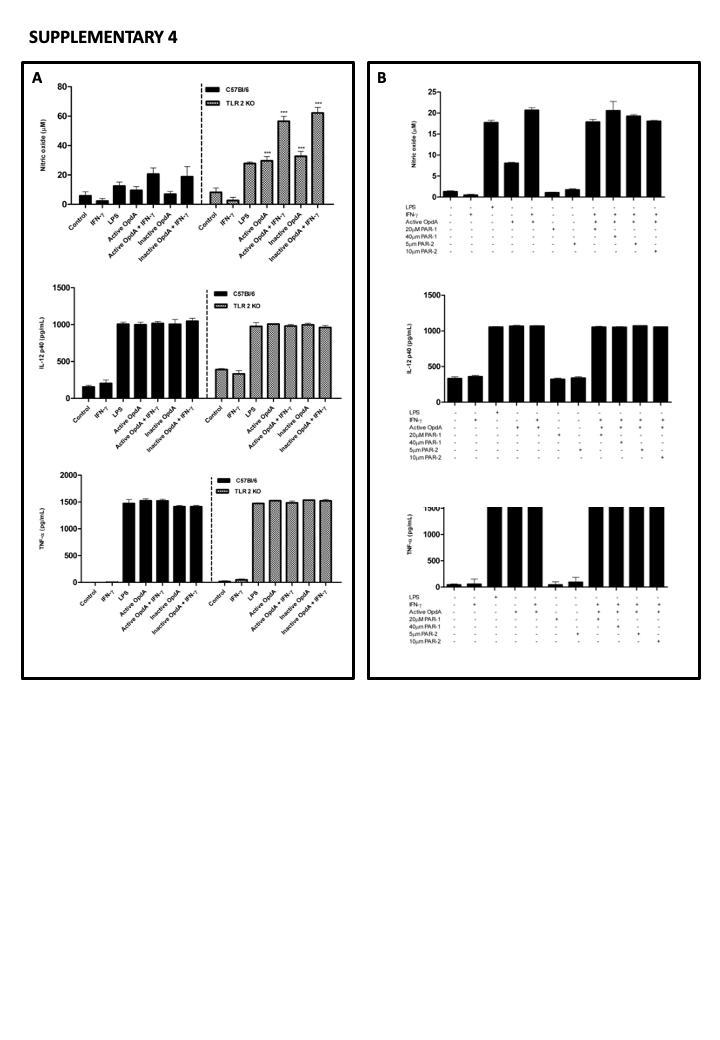

Supplement: Supplementary Figure 4 — APC activation by active or inactive rOpdA does not depend on TLR2, PAR-1, or PAR-2. (A) APCs from TLR2-/- and C57Bl/6 mice were incubated in U-bottom plates (1x10^5^ cells/well) and then stimulated with active or heat-inactivated rOpdA (50 µg/mL), LPS (200 ng/mL), and IFN-γ (200 U/mL) for 48 hours. NO was measured in the culture supernatants using Griess reagent, and IL-12 (p40) and TNF-α levels were determined by ELISA. (B) APCs from C57Bl/6 mice were incubated as described and pretreated for 1 hour with the reverse peptides of PAR-1 (5 or 10 µM) or PAR-2 (20 or 40 µM). Subsequently, these cells were stimulated with LPS (200 ng/mL), IFN-γ (200 U/mL), and active rOpdA (50 µg/mL). The secretion of NO, IL-12(p40), and TNF-α was analyzed by ELISA. Results are shown as the average ± SD from triplicate samples. Data from one of two independent experiments are presented. *** p < 0.0001, analyzed by two-way ANOVA with Bonferroni post-test, comparing C57Bl/6 mice with the same stimulus. [file Image4.tiff]
